# The emerging role of regulatory cell-based therapy in autoimmune disease

**DOI:** 10.3389/fimmu.2022.1075813

**Published:** 2022-12-14

**Authors:** Farbod Ghobadinezhad, Nasim Ebrahimi, Fatemeh Mozaffari, Neda Moradi, Sheida Beiranvand, Mehran Pournazari, Fatemeh Rezaei-Tazangi, Roya Khorram, Maral Afshinpour, Rob A. Robino, Amir Reza Aref, Leonardo M. R. Ferreira

**Affiliations:** ^1^ Student Research Committee, Kermanshah University of Medical Sciences, Kermanshah, Iran; ^2^ Universal Scientific Education and Research Network (USERN) Office, Kermanshah University of Medical Sciences, Kermanshah, Iran; ^3^ Division of Genetics, Department of Cell and Molecular Biology and Microbiology, Faculty of Biological Science and Technology, University of Isfahan, Isfahan, Iran; ^4^ Department of Nutrition, School of Medicine, Zabol University of Medical Sciences, Zabol, Iran; ^5^ Division of Biotechnology, Department of Cell and Molecular Biology and Microbiology, Nourdanesh Institute of Higher Education, University of Meymeh, Isfahan, Iran; ^6^ Department of Biology, Faculty of Basic Sciences, Islamic Azad University, Shahrekord, Iran; ^7^ Clinical Research Development Center, Imam Reza Hospital, Kermanshah University of Medical Sciences, Kermanshah, Iran; ^8^ Department of Anatomy, School of Medicine, Fasa University of Medical Sciences, Fasa, Iran; ^9^ Bone and Joint Diseases Research Center, Department of Orthopedic Surgery, Shiraz University of Medical Sciences, Shiraz, Iran; ^10^ Department of Chemistry and Biochemistry, South Dakota State University, Brookings, SD, United States; ^11^ Department of Microbiology and Immunology, Medical University of South Carolina, Charleston, SC, United States; ^12^ Department of Regenerative Medicine and Cell Biology, Medical University of South Carolina, Charleston, SC, United States; ^13^ Hollings Cancer Center, Medical University of South Carolina, Charleston, SC, United States; ^14^ Department of Medical Oncology, Dana-Farber Cancer Institute, Harvard Medical School, Boston, MA, United States; ^15^ Xsphera Biosciences, Boston, MA, United States

**Keywords:** autoimmunity, regulatory immune cells, immune suppressive cells, adoptive cell therapy, regulatory T cell, tolerogenic dendritic cell, IL-10-producing cell, regulatory B cell

## Abstract

Autoimmune disease, caused by unwanted immune responses to self-antigens, affects millions of people each year and poses a great social and economic burden to individuals and communities. In the course of autoimmune disorders, including rheumatoid arthritis, systemic lupus erythematosus, type 1 diabetes mellitus, and multiple sclerosis, disturbances in the balance between the immune response against harmful agents and tolerance towards self-antigens lead to an immune response against self-tissues. In recent years, various regulatory immune cells have been identified. Disruptions in the quality, quantity, and function of these cells have been implicated in autoimmune disease development. Therefore, targeting or engineering these cells is a promising therapeutic for different autoimmune diseases. Regulatory T cells, regulatory B cells, regulatory dendritic cells, myeloid suppressor cells, and some subsets of innate lymphoid cells are arising as important players among this class of cells. Here, we review the roles of each suppressive cell type in the immune system during homeostasis and in the development of autoimmunity. Moreover, we discuss the current and future therapeutic potential of each one of these cell types for autoimmune diseases.

## Introduction

Protecting the body against foreign pathogenic agents and activating repair systems when tissue is damaged are the primary functions of our immune system. The immune system must constantly strike a balance between attacking harmful agents and preventing damage to the body. Disturbances in this balance can cause autoimmune diseases, in which the immune system attacks self-tissues. Although investigations have focused more on the role of adaptive immunity, both innate and adaptive immunity appear to be involved in the development of autoimmune diseases (1). Albeit individually rare, autoimmune disorders comprise a wide range of complex diseases that affect about 5% of the world’s population. Loss of self-tolerance leads to the production of autoantibodies against self-tissues and cells, as well as the emergence of autoreactive T cells (2). The incidence of autoimmune diseases is increasing worldwide (3). Within these, type 1 diabetes (T1D), multiple sclerosis (MS), systemic lupus erythematosus (SLE), rheumatoid arthritis (RA), and Crohn’s disease (CD) are the most common types of autoimmune diseases, posing enormous health challenges (4). Currently, the main goal of autoimmune disease research is to find more effective treatment solutions. Present treatments can alleviate some autoimmune symptoms but lack specificity and need to be prescribed for long periods. In contrast, the use of living drugs, such as regulatory T (Treg) cells, a subset of T lymphocytes dedicated to inhibiting specific immune responses, holds the potential to be more specific, cause less side effects, and be more effective (5). Current drugs used to treat autoimmune disease include immunosuppressive drugs, such as ciclosporin (cytokine gene transcription inhibitor), anti-metabolite drugs, such as azathioprine (purine synthesis inhibitor), and biologic drugs, such as belimumab (human monoclonal antibody against B-cell activating factor). Cell therapy as another approach for treat autoimmune disease is assumed that are less toxic and can aim to help restore immune tolerance, as opposed to global non-specific immune suppression. Therefore, checking the accuracy of these assumptions is on the agenda of many ongoing studies (5). Notable examples of such living drugs include immune cells, such as regulatory B (Breg) and Treg cells. In general, the superior ability of immune cells, when compared to small molecules and biologicals, to either maintain or disrupt the body’s immune balance provides a unique opportunity to treat autoimmune disorders and accelerate the repair of deregulated nodes in the immune system (4). Many studies have been performed on cell-based therapy for various types of autoimmune diseases. Several types of immune cells, including Breg and Treg cells, regulatory dendritic cells (DCs), as well as mesenchymal stem cells, monocytes, and macrophages, have been shown to relieve inflammation and symptoms of autoimmune diseases (6, 7). Adoptive immune cell therapies have sparked great interest due to their advantages, including delivery convenience, capacity of naturally homing to target tissues, and the ability to significantly alter the course of disease, as supported by preclinical research and promising results from early clinical trials in autoimmune diseases and transplantation (8). Here, we review a variety of immune cell-based therapies for autoimmunity, including their prospects and potential for treating and reducing the burden of these diseases worldwide.

## Regulatory T cells

A subset of naïve T cells develops naturally in the immune system to maintain immune homeostasis and autoimmune tolerance (9). During 1970-1980, many efforts were made to detect CD4+ T cells able to suppress autoimmune diseases in rodents through reliable molecular markers (10). Finally, in the mid-1990s, it became clear that this group of CD4^+^ T cells, known as regulatory T cells (Treg cells), continuously express the α chain of the interleukin 2 receptor (IL-2), or CD25, at high levels (11). CD4^+^CD25^+^ cells comprise only 3-10% the peripheral CD4^+^ T cell population. Their key role in autoimmune diseases was clarified when their deletion was shown to result in a wide range of human-like autoimmune disorders in healthy mice, including type 1 diabetes, thyroiditis, and autoimmune gastritis. On the other hand, infusion of CD4^+^CD25^+^ T cells inhibited autoimmune disease development in mice (12). In 2003, it was reported that CD25^+^CD4^+^ T cells in rodents and humans uniquely express the transcription factor Foxp3 at high levels (13). Mutations in the Foxp3 gene in both mice and humans cause autoimmune and inflammatory diseases such as type 1 diabetes (T1D) and thyroiditis. In addition, the incidence of allergy and inflammatory bowel disease (IBD), which together cause IPEX syndrome (immune dysregulation polyendocrinopathy enteropathy X-linked (IPEX) syndrome), is caused by dysfunction in FOXP3^+^ T cells (14). In summary, regulatory T cells develop in the thymus, are characterized by high constitutive expression of Foxp3 in the nucleus and CD25 on the surface, and suppress excessive immune responses against environmental, microbial, and self-antigens (15).

**Figure 1:**
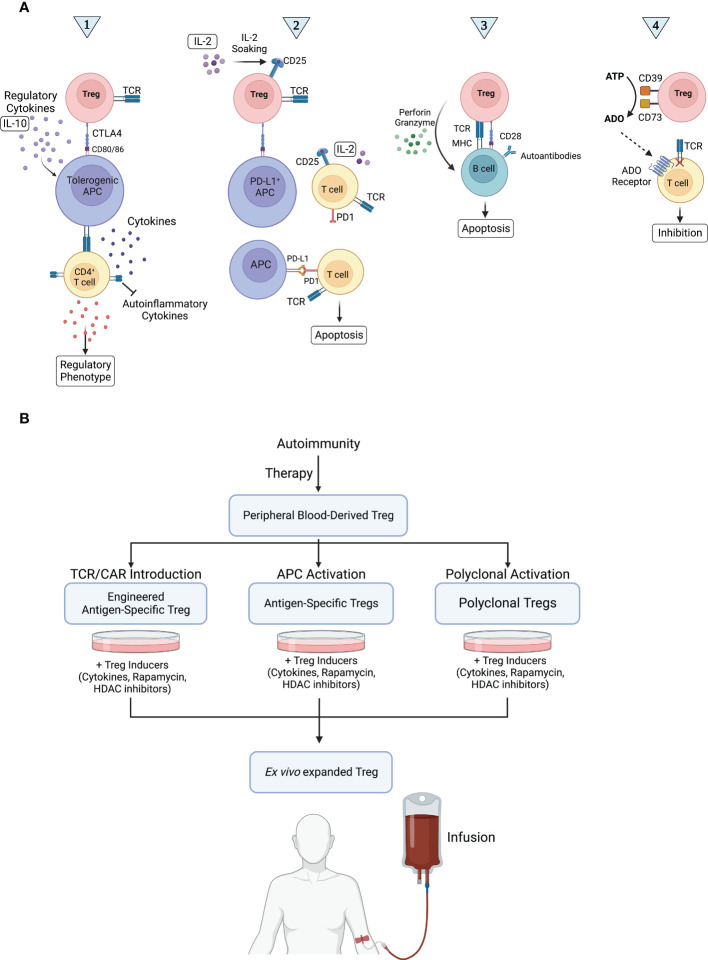
Treg cell mechanisms of action in autoimmunity and therapeutic application. **(A)** Treg cells are thought to work *via* four main routes. Route 1: Treg cells expand and secrete large amounts of IL-10, enhancing tolerogenic APC activity. Tolerogenic APCs in turn interact with CD4^+^ T cells, inhibiting their release of self-inflammatory cytokines. In addition, tolerogenic APCs stimulate the development of a regulatory phenotype in naïve T cells and induce the production of Treg cells, ultimately suppressing autoimmune reactions. Route 2: The release of IL-2 in the microenvironment is detected by CD25^+^ Treg cells, which interact with and induce PD-L1 expression in APCs. PD-L1^+^ APCs then induce apoptosis in activated PD-1^+^ Teff cells *via* PD-1/PD-L1 signaling, inhibiting immune responses, such as those targeting self-antigens. Route 3: The interaction of Treg cells with autoreactive B cells induces B cell apoptosis *via* perforin/granzyme-mediated cytotoxicity, preventing autoantibody production. Route 4: Treg cells convert extracellular ATP into adenosine (ADO), a potent immunosuppressant, using ectoenzymes CD39 and CD73. ADO binds to its receptor in Teff cells, inhibiting them. **(B)** Any disruption in routes 1, 2, 3, and 4 can lead to autoimmunity. In Treg cell-based therapy, Treg cells are collected from peripheral blood, activated, and expanded *ex vivo*. Finally, Treg cells are injected to suppress immune responses *via* routes 1, 2, 3, and 4.

Treg cells constitute a form of dominant tolerance, directly suppressing the activation, expansion, and function of effector immune cells. A number of mechanisms of Treg cell-mediated immune suppression have been revealed (**Figure 1A**), from depletion of IL-2 in the milieu *via* high surface expression of CD25 and secretion of anti-inflammatory cytokines, such as IL-10, IL-35, and TGF-β (contact-independent) to trogocytosis of CD80 and CD86 receptors in antigen presenting cells (APCs) *via* CTLA4 (contact-dependent) (16, 17). Treg cell function is classically characterized by two main tenets: infectious tolerance and bystander suppression. Infectious tolerance consists of the transfer of suppressive capacity from one cell population to another and is believed to occur mainly *via* anti-inflammatory cytokines that block dendritic cell (DC) maturation, bestowing DCs with a tolerogenic phenotype and drive naïve T cell differentiation into induced Tregs (18, 19), whereas bystander suppression refers to Tregs’ capacity to suppress immune responses specific for an antigen distinct from the one they recognize in the same milieu (20). Both phenomena are local, happening at the level of the immune microenvironment.

Despite these hallmarks, heterogeneity of the Treg cell compartment is one of the main challenges of studying Tregs in autoimmune diseases and at large. Systematic characterization of Treg cell subsets can lead to a more precise identification of their roles in various autoimmune diseases and hence the development of more specific treatment strategies for treatment (21).

## Types of regulatory T cells

Treg cells can be divided based on their developmental origin. Thymic Tregs (tTreg) develop in the thymus. A small fraction of Treg cells is derived from conventional T cells (Tconv) and mature peripherally (pTreg) under certain conditions, including exposure to microbial antigens in the intestinal mucosa. Both tTregs and pTregs express Foxp3 and have suppressive function. On the other hand, *ex vivo* antigenic stimulation in the presence of cytokines TGF-β and IL-2 can induce Foxp3 expression in Tconv cells, which are phenotypically and functionally similar to tTreg and pTreg cells and are known as induced Treg cells (iTreg). However, iTreg cells differ in terms of functional stability and specificity and, therefore, come with different advantages and challenges in treating autoimmune diseases (22).

Type 1 regulatory T (Tr1) cells are a subtype of induced regulatory cells. Unlike Treg cells, Tr1 cells secrete immunosuppressive cytokines, such as IL-10 and TGF-β, but do not express CD25 or Foxp3. In addition, Foxp3^+^ Treg cells and Tr1 cells differ in their metabolic programs (23). For example, *in vitro* induced Tr1 cells display high rates of aerobic glycolysis, while Foxp3^+^ Treg cells prefer oxidative phosphorylation. Although both Foxp3^+^ Treg cells and Tr1 cell populations are present in the spleen, Peyers’ patches, and lymph nodes, Tr1 cells are more abundant in the small intestine, whereas Foxp3^+^ Treg cells are more abundant in the large intestine. Interestingly, mice lacking induced Foxp3^+^ Treg cells have a compensatory increase in Tr1 cells in the mesenteric lymph nodes (24). Transfer of Foxp3^+^ Treg cells, on the other hand, results in the generation of Tr1 cells and the development of antigen specific tolerance in recipient mice in an allogeneic pancreatic islet transplantation model. Overall, it appears that Foxp3^+^ Treg cells are essential for initiating tolerance induction at the site of inflammation, while Tr1 cells may be important for the long-term maintenance of immune tolerance in some settings (25). Consistent with a role for Tr1 cells in immune tolerance, Tr1 cell dysfunction has been reported in autoimmune patients, which also indicates their therapeutic potential. However, a high dose of Tr1 cells is needed to be effective in treatment. A Phase I trial (NCT03198234) testing a cell product, T-allo10, containing up to 15% CD49b^+^LAG-3^+^ Tr1 cells, is administered during HLA mismatched hematopoietic stem cell transplant (HSCT). T-allo10 is created by stimulating donor-derived CD4^+^ T cells with host-derived tolerogenic DCs (DC-10) in the presence of IL-10. The therapy is well tolerated and T-allo10 cells detectable in the peripheral blood of patients up to 1 year after transfer, but the effects on GvHD and long-term tolerance are still being studied (26).

Treg cells can also be classified based on their function. Curiously, comprehensive analysis of human peripheral blood Treg cells revealed the existence of subsets of T helper-like Treg cells, *i.e.* Treg cells that share chemokine receptor and transcription factor expression with T helper cells while being suppressive. For instance, Th1-Treg cells can be characterized as CXCR3^+^T-BET^+^FOXP3^+^ Treg cells, Th2-Treg cells as CCR8^+^GATA3^+^FOXP3^+^ cells, and-so-forth (27–29). Recent work by Levings and colleagues showed that it is possible to generate Treg cells *in vitro* that preferentially migrate to Th1-inflamed sites (30). In this study, interferon-gamma (IFN-γ) and IL-12 were added during Treg cell expansion *in vitro*, resulting in epigenetically stable Th1-like CXCR3^+^T-BET^+^FOXP3^+^ Treg cells (30). In a separate study, investigators discovered that a larger proportion of activated FOXP3^hi^CD45RA^lo^ Treg cells in allogeneic hematopoietic stem cells was associated with less development of acute GvHD in bone marrow transplant patients (31). Utilizing tissue-specific and/or Th-like Treg cell subsets (29, 32) in therapies for autoimmune disorders requires more research. Yet, one can envision specific subsets of Treg cells being advantageous in scenarios where certain cytokine secretion patterns or tissue homing properties are instrumental for treatment efficaciousness.

Another category of Th-like Treg cells is follicular regulatory T (Tfr) cells, which inhibit follicular helper T (Tfh) cells. Tfr cells were first identified in mice and play a vital role in the germinal center response and antibody production (33). Tfr cells highly express CXCR5, and, similarly to Tfh cells, require CD28 and ICOS for their development and maintenance (34). In addition, while Tfh cells are derived from CD4^+^Foxp3^-^ precursors, Tfr cells originate from CD25^+^Foxp3^+^ precursors. Recent studies have also shown that deficiencies in Tfr cells lead to antibody accumulation and the occurrence of a wide range of autoimmune diseases (35). Interestingly, when compared to healthy donors, coronavirus disease 2019 (COVID-19) convalescent patients with severe disease, which has been hypothesized to have an autoimmune-like component, had higher frequencies of effector memory Tfh cells and lower frequencies of central memory Tfh cells (36).

## Using Treg cells to treat autoimmunity

Based on the characteristics of the different subsets of Treg cells, different strategies can be adopted for the treatment of autoimmune diseases using them (**Figure 1B**). So far, results of treatments with Treg cells in the early stages of clinical trials for patients with transplant rejection, GvHD, and autoimmune disorders have been promising (37). However, one of the main challenges of these studies is the isolation of pure Treg cells and their expansion to an adequate amount for clinical applications, to reach the so-called clinical dose. Treg cell therapy is based on the idea that injecting of an efficient dose of Treg cells restores the balance between effector T cells and immune-regulatory cells (such as Treg cells) in favor of increasing immune tolerance. Some clinical studies have shown that Treg cell treatment can effectively and safely reduce autoimmune symptoms and organ transplant rejection (38). The first human clinical study for adaptive Treg transfer was reported by Trzonkowski and colleagues in 2009. Treg cells (CD4^+^CD25^+^CD127^-^ cells) extracted from two donor families were expanded *ex vivo* and transferred to GvHD patients. Infusion of Treg cells significantly reduced disease symptoms in subjects with chronic GvHD and resulted in immunosuppression (39). However, in grade 4 acute GvHD, symptoms improved only temporarily. Recently, the adoptive transfer of Treg cells to SLE patients was investigated by Dall’Era and colleagues. Flow cytometry and whole transcriptome analyses revealed that accumulation of Treg cells in the skin subdued IFN-γ pathway and increased IL-17 pathway activity. This group reported the first case of adoptive transfer of Treg cells to SLE patients. In general, their results showed that this treatment led to an increase in Treg cells in the inflamed skin and a dynamically change in local immune response from Th1 to Th17 (40). Achieving good results using Treg cell adoptive transfer into patients requires methods to isolate and expand Treg cells from different sources with high efficiency and purity. In addition, there has been uncertainty about how well *in vitro* functional assays correlate with *in vivo* activity, and the complexity of modifying protocols to improve properties such as antigen specificity and homing receptor expression.

## Sources of Treg cells for extraction and expansion

Treg cells are distributed almost throughout the body; peripheral blood and umbilical cord blood (UCB) are the most valuable sources for Treg cell isolation. Currently, the most common source for the production of autologous Treg products is peripheral blood. Yet, with the increased prevalence of cord blood banking, UCB may eventually become a more abundant and common source for autologous Treg cells (41). Indeed, using third-party UCB units as an allogeneic source of Treg cells for therapy is becoming more common. These products are enriched with naïve Treg cells, which have the potential to expand more than memory cells, with each UCB unit containing *ca.* 6 x 10^6^ such cells (42). According to protocols laid out by Brunstein and colleagues, this number can be expanded *in vitro* more than 27,000 times to reach a clinical dose (43).

The pediatric thymus is another source of Treg cells that has recently received attention. Usually, the thymus is removed during pediatric cardiac surgery (44). The main advantage of using pediatric thymus is the high number of Treg cells in this organ, 100 times more than in a UCB unit, or about 500 x 10^6^ cells. Furthermore, the almost complete absence of Tconv cells in the pediatric thymus has made it easier to purify Treg cells, significantly reducing the possibility of contamination with Tconv cells. Interestingly, thymus Treg cell (tTreg) immunosuppressive function is greater than that of Treg cells derived from peripheral blood or UCB, even in the presence of inflammation. Among other advantages of tTreg cells, it can be mentioned that they express an especially high level of CD25, which leads to high sensitivity to IL-2 stimulation even at low doses (45). As IL-2 also drives the activation of Tconv and natural killer cells (NK cells), stimulating a robust inflammatory response, it is advantageous that tTreg cells can be stimulated and expanded with IL-2 in levels at which Tconv cells and NK cells do not respond (46).

Other Treg sources include non-lymphatic tissues, namely intestines, lungs, joints, skin, and muscles. Although the purification methods for these Treg cells are laborious and low efficiency, efforts to expand tissue Tregs in the laboratory continue due to their unique characteristics (47). In parallel, efforts are underway to differentiate induced pluripotent stem cells (iPSC) into Tregs, as well as to convert Tconv cells into iTreg cells by inducing Foxp3 expression (48). For example, ectopic Foxp3 expression together with Notch signaling pathway activation by stromal cells made it possible to produce Treg cells from mouse iPSCs. This approach will most likely be used in gene therapy for IPEX (49). Alternatively, iTreg cells can be generated from CD4^+^CD25^-^ Tconv cells in the presence of IL-2 and TGF-β. Currently, iTreg cells are in clinical trials (NCT 01634217) (50).

## Comparing autologous with allogeneic Treg cells

A critical point in cell therapy design is deciding whether to use autologous or allogeneic cells. With regards to autoimmune diseases and organ transplantation, all clinical trials thus far have used the patient’s autologous peripheral blood Treg cells. In contrast, allogeneic cells are commonly derived from UCB units with at least 4 HLA alleles in common with the recipient (51, 52). Although autologous Treg cells have the highest chance of being accepted by the recipient, their production can be very challenging. Autologous products must be manufactured uniquely for each patient, so manufacturing processes must be robust and reproducible despite the high variability between donors. On the other hand, the cost of producing autologous cells for each patient is very high; for this reason, attention has been drawn to allogeneic cell therapy products (51, 52). In addition, allogeneic cells can be derived from primary cell populations with less variability and, as a result, are more comprehensively under quality control to reduce patient risks. Studies on animal models have shown that allogeneic Treg cells have the same power as donor-derived Treg cells in preventing graft rejection (53). In humans, UCB-derived Treg cells were safe and reduced the incidence of acute and chronic GvHD in transplant patients (51, 52). Although allogeneic cell therapy products have only been investigated in immunocompromised people, their main limitation of allogeneic cells in healthy people is limited survival time due to rejection in the patient (54). In non-human primates, allogeneic Treg cells in the blood could not be detected by flow cytometry for more than 3 to 6 weeks after infusion (54). On the other hand, autologous Treg cells can be identified by mass spectrometry for more than a year after injection. Using a flow cytometry-based readout, the percentage of Tregs in circulation peaked 7-14 days post-transfer and fell near the detection limit within three months, similar to findings with allogeneic cells (40, 55). As a result, more research is needed to determine why the majority of infused Tregs appear to disappear from circulation in these various contexts.

One of the challenges is not determining the tolerance level for HLA mismatch in Treg cells. In studies of antiviral T therapy, third-party virus-specific T cells with the lowest HLA mismatch (one allele) reduced viral load, which can be exploited for the treatment of drug-resistant infections after hematopoietic stem cell transplantation (56). These results suggest that it is not necessary to fully match the HLAs to effectively transfer Treg cells. Of note, if the suppressive function of Treg cells is transferred to other cell populations, *i.e.* infectious tolerance, there is no need for the long-term survival of Treg cells (57). Allogeneic Treg products pose a risk of alloimmune sensitization, especially in GvHD patients and non-immunosuppressed patients. However, treatment with allogeneic specific Treg cell transfer after allogeneic hematopoietic stem cell transplantation did not lead to severe GvHD (58). Treg cells are generally less prone to allogeneic sensitization due to their role in suppressing the immune response compared to Tconv cell transfer. To increase the probability of allogeneic Treg cell survival in the patient, genetic modification can be used to remove or edit HLA molecules. Research in the regenerative medicine and stem cell field is driving the field of HLA editing, featuring strategies such as knocking out β-2 microglobulin to eliminate HLA class I surface expression) and/or CIIT2 to eliminate HLA class II (59). As such changes can render the cells susceptible to NK cell-mediated lysis, complementary strategies seek to increase the expression of non-classical HLA molecules to inhibit NK cells (59, 60).

## Comparing polyclonal with antigen-specific Treg cells

Selecting between polyclonal Treg cells or antigen-specific Treg cells is another decision to be made when designing Treg cell therapies. Although producing polyclonal Treg cells requires less effort, a large number of them need to be injected for their effectiveness. On the other hand, only a small fraction of Treg cells is required for antigen-specific Treg cell strategy (61). Using antigen-specific Treg cells are reduced off-target suppression and increased potency. Putnam and co-workers expanded alloreactive Treg products by co-culturing recipient Treg cells with donor B cells (62). Clinical studies with alloreactive Tregs focus on solid organ transplantation and GvHD, namely NCT02711826, NCT01795573, NCT02188719, and NCT02244801.

To circumvent the rarity of antigen-specific Treg cells, methods of artificially generating antigen-specific Treg cells have been developed, including ectopic expression of a chimeric antigen receptor (CAR) or a T cell receptor (TCR), as discussed in later sections. TCR recognition is HLA-restricted and TCR affinity towards its cognate antigen is lower than that of CARs. Moreover, as recognition of antigens by CARs is not limited to HLA, CAR Treg cell therapy can target more diverse antigens, including protein, carbohydrate, and glycolipid antigens (63).

## Generating Treg cells for adoptive transfer

CD25 is present on all Treg cells, independently of their origin, and is a selective marker for Treg cell isolation (64). Instead of relying on CD25 expression alone, fluorescence-assisted cell sorting (FACS) CD45RA^+^CD25^+^CD127^-^ naïve Treg cells increases Treg purity (65). Compared to magnetic selection, closed-system Good Manufacturing Practice (GMP) compatible FACS can simplify Treg cell isolation based on multiple markers. Miltenyi’s three-laser MACSQuant Tyto Cell Sorter allows for GMP-compatible FACS isolation of human Treg cells by utilizing single-use closed-cartridge systems, preventing contamination between samples and aerosol formation. Following isolation, Treg cells are expanded *in vitro* to reach clinical dose. Bead-immobilized antibodies, artificial antigen-presenting cells (APCs), and soluble antibody reagents are standard methods used to activate Treg cells *in vitro*.

Many Treg expansion protocols use magnetic beads covalently attached to anti-CD3 and anti-CD28 antibodies. However, removing the magnetic beads before infusion has limited their application due to cell loss and potential incomplete bead removal. With regards to artificial APCs, co-stimulatory molecules and an Fc receptor are expressed on the cell surface. Studies have shown that K562 cells expressing the co-stimulatory receptor CD86 and the high affinity Fc receptor CD64 and loaded with anti-CD3 antibody perform better than anti-CD3/CD28 beads to expand UCB Treg cells. K562 is a human myelogenous leukemia cell line that is devoid of HLA and CD80/CD86 and can be readily expanded. By loading these cells with anti-CD3 monoclonal antibody, the primary signals for Treg cell activation are triggered through binding to anti-CD3 sequestered by CD64 and to CD86. Because these artificial APCs are lethally irradiated, they gradually disappear from culture, obviating the need to eliminate them before injection (66). However, while artificial APCs are particularly successful in stimulating Treg growth, they complicate the cell production process by necessitating additional cell testing and batch validation. For ease of elimination before Treg administration to the patient, additional activation reagents are available in the soluble form. For instance, the T cell TransAct is a nanomatrix polymer conjugated to anti-CD3 and anti-CD28 antibodies. Since this reagent is soluble, it is easily removed by centrifugation (67). However, its use in clinical studies has not yet been reported. Overall, culture and expansion protocols for Treg cells for clinical use vary, with several research groups working towards reaching maximum efficiency (38).

Another critical point in the design of cellular therapy is the choice between fresh or cryopreserved Treg cells. Since cryopreservation provides several advantages, including the possibility of long-term storage of the product, increased time for injection, and hence more time for release testing, many researchers have turned to it. However, the reduction in the quality of Treg cells after cryopreservation is an important limitation of choosing this strategy for clinical applications. Studies on the decline of the quality of FOXP3^+^ Treg cells and CD25^high^CD127^-^ Treg cells after cryopreservation have produced contradictory results (68). Although it has been reported that the expression of Foxp3, CD25, and the suppressive activity of Treg cells decreases after thawing, these characteristics can be restored after reactivation (69). Yet, few clinical trials using cryopreserved Treg cells have been reported due to uncertainty regarding the effects of freezing and thawing these cells (70). This and other parts of the Treg manufacturing process stand to benefit from advances in the production of Tconv cells for cancer immunotherapy (71).

## Increasing the stability and efficiency of Treg cells

One factor determining the effectiveness of Treg cells is their successful migration to both inflammation sites and lymph nodes (72). Therefore, many researchers are looking for protocols that, in addition to maximizing Treg stability, also increase their migration potential, which requires increasing the expression of homing receptors. For example, Hoeppli and colleagues reported that, by creating appropriate cell culture conditions, stable expression of homing receptors such as α4β7 and CXCR3 chemokine receptor can be induced (30). INF-γ and IL-2 addition during Treg cell expansion increased the expression of CXCR3, enabling Treg cell migration towards CXCL10 (30). Importantly, these cells maintained high expression levels of CXCR3 after injection into mice and in the absence of INF-γ and IL-2. Concomitant expression of CD62L and CCR7 further bestowed these cells will the potential to migrate to lymphoid tissues and the site of inflammation. Interestingly, Parmer and colleagues found that adding fucose to the surface of Treg cells induces sialyl-Lewis X moiety formation on the P-selectin ligand, increasing the viability of UCB Treg cells in GvHD likely by increasing the binding potential to E-selectin (73). Indeed, this strategy is being investigated in the NCT 02423915 phase I/II clinical trial, which assesses the effectiveness and safety of fucosylated Treg cells in reducing or preventing GvHD in humans.

In addition to optimizing culture conditions, two other strategies, creating antigen-specific Treg cells and increasing the expression of Treg-specific genes, can improve Treg cell function. For over a decade, studies have shown that antigen-specific Treg cells are more potent than their polyclonal counterparts. Infusing Treg cells with donor allospecific TCRs leads to long-term survival in mice with MHC-mismatched heart transplants (74). This notion is currently being utilized in the context of autoimmunity, where the restricted number of disease peptide-MHC complexes allows for the selection of a few TCRs to engineer Tregs compatible with a substantial proportion of afflicted people (75). One of the alternative approaches to TCRs is the engineering of Treg cells to express a CAR specific for the desired antigen. CARs are synthesized proteins with combined extracellular antigen binding domains, so-called single chain fragment variable (scFv), and intracellular signaling domains (63, 76). Preclinical studies in mice have demonstrated the promise of this strategy. For example, in the treatment of experimental autoimmune encephalomyelitis (EAE), a mouse model of multiple sclerosis, CAR Treg successfully targeted the myelin glycoprotein of oligodendrocytes and ameliorated disease. Similar results were obtained in the treatment of colitis by targeting carcinoembryonic antigen (CEA). Further studies showed that human HLA-A2-specific CAR Treg cells perform better than polyclonal cells in preventing xenogeneic GvHD (77, 78).

## Treg cell-targeted therapy for autoimmune disease

Our increasing understanding of the role of Treg cells in autoimmune diseases has made Treg cell-targeted therapies promising strategies to alleviate these disorders. Two such strategies, low-dose interleukin-2 (IL-2) administration and Treg cell adoptive transfer, have received much attention in recent research and have been tested in clinical trials for several autoimmune diseases (40, 51, 52, 55, 79–82) (**Table 1**).

**Table 1:** Recent clinical studies on Treg cell therapy in autoimmune disease.

Autoimmune disease	Treg therapy	Study	Patient No.	Comedications	Treg results	Refs./Study ID
Crohn’s Disease	ova-Tregs isolated from PBMCs Single infusion Tr1 intravenously	Phase I/IIa	20 patients	–	Safe, unknown efficiency, well tolerated,	(83)
Crohn’s Disease	ova-Tregs, infusion Tr1 intravenously	Phase I/II	32 participants	Ovasave	Well tolerated	NCT02327221
Crohn’s Disease	Polyclonally expanded CD4+CD25+CD127lowCD45RA+Tregs, Intravenously	Double-blind, placebo-controlled trial	24 participants	TR004	Suppression of activation of lamina propria and mesenteric lymph node lymphocytes	NCT03185000
Type 1 Diabetes	Administration of autologous expanded (ex vivo) Tregs	Phase I	12 DM1 children		Safe and feasible, lower requirement for exogenous insulin. No adverse effects	(79)
Type 1 Diabetes	Administration of autologous expanded Tregs (ex vivo) (CD4^+^CD127^lo/−^CD25^+^ polyclonal Tregs)	Phase I	14 Patients		Safe and feasible. Retention of CD4^+^CD25^hi^CD127^lo^FOXP3^+^Treg cells	(55)
Type 1 Diabetes	A single infusion of CLBS03 Low Dose, a cell product comprised of autologous, ex vivo expanded regulatory T-cells resuspended in sterile infusion solution.	Phase II	113 participants		Not Recruiting	NCT02691247
Type 1 Diabetes	Administration of autologous expanded polyclonal Tregs (ex vivo) single dose of CD4^+^CD25^+^CD127^low^ cells and low-dose IL-2	Phase I			Completed. Low-dose IL-2 expands exogenously administered Tregs	NCT02772679 (84)
Type 1 Diabetes	Administration of umbilical cord blood Tregs combined to insulin	Open-label phase I/II	40 participants		More effective than insulin therapy alone. Recruiting	NCT02932826
Active Cutaneous Lupus	Administration of autologous expanded Tregs (ex vivo) single dose	Phase I	1 participants		Terminated due to participant recruitment feasibility	NCT02428309
Active Cutaneous Pemphigus	Infusion of a single dose autologous polyclonally expanded Tregs	Open-label phase I	5 participants		Active, not recruiting	NCT03239470
Autoimmune Diabetes	Umbilical cord blood Tregs combined to liraglutide therapy	Phase I/II	40 participants		Recruiting	NCT03011021
Autoimmune Hepatitis	Infusion of a single dose autologous polyclonally expanded Tregs	Open-label phase I/II	30 participants		Not yet recruiting	NCT02704338
Systemic Lupus Erythematosus	Treg adoptive cell therapy	Clinical study	1 participant		Increased activated Tregs in inflamed skin with a dynamic shift from Th1 to Th17 responses	(40)
Inflammatory Bowel Disease	Single infusion of regulatory Treg cells (type 1 ovalbumin-specific)	Phase I/IIa clinical study			Well tolerated, dose-related efficacy	(85)
Pemphigus Vulgaris	Polyclonal autologous Treg cells therapy	Phase Ia multicenter clinical trial	5 participants		Active, not recruiting	NCT03239470

PBMCs, patients’ peripheral blood mononuclear cells; ova-Tregs, Ovalbumin-specific Treg cells.

## Treg cell adoptive transfer

In its current form, Treg cell adoptive transfer strategy requires large Treg cell numbers to be clinically effective, in the order of 8 x 10^9^ cells. For this purpose, Treg cells are collected from autologous peripheral blood (40, 79) or umbilical cord blood (51, 82) and expanded *ex vivo* using a number of protocols. As mentioned before, recent research points toward artificial APCs as key in increasing the number of tTreg cells obtained. In addition, FOXP3 expression and the suppressive function of tTreg cells are maintained by artificial APCs (86–88). Other research has shown the role of various biological compounds, including retinoic acid, IL-2, rapamycin, TGF-β (transforming growth factor-β), histone deacetylase (HDAC) inhibitors, DNA methyltransferase inhibitors, among others, in supplying Treg cells with functional and phenotypic stability (89–91). For instance, a study by Lu and colleagues determined that tTreg cells displayed higher expansion and suppressive function *in vitro* and *in vivo* following antagomir-mediated knockdown of miR-146b-5p (92). Another study elucidated the role of D-mannose in inducing FOXP3 expression and the conversion of naïve T cells into Treg cells (93). In addition, a systematic review by Dwivedi and colleagues indicates that prebiotics (substrates that support colonization by specific non-pathogenic bacteria) and probiotics (non-pathogenic bacteria or bacterial products found in food supplements) have a significant effect on the proliferation and induction of Treg cells in animal models and human cell cultures (94). More recently, Skartsis and colleagues showed that culturing human polyclonal Treg cells *ex vivo* in the presence of IL-6 and tumor necrosis factor alpha (TNF-α), two pleiotropic cytokines, in conjunction with CD28 super-agonist, dramatically boosts their proliferation while maintaining their phenotype and suppressive function *in vitro* and *in vivo* (95). Altogether, these and ongoing investigations provide practical methods to continue improving on the generation of adequate numbers of *bona fide* human Treg cells for adoptive cell therapy for the treatment of autoimmune diseases.

Treg cell adoptive therapy has been tested in various autoimmune diseases (5, 96). For instance, while SLE is characterized by unusual innate and adaptive immune responses, mounting evidence shows the essential role of Treg cells in this complex autoimmune disease, especially in its peak stages (97–100). In preclinical studies, Treg cell infusion in autoantibody-positive mice delayed renal complications and significantly increased their survival rate (101). In a case study reported by Dall’Era and colleagues, autologous Treg cells were amplified and administered to one SLE patient. The authors observed an increased percentage of activated Tregs in diseased skin (40). In phase I trials focused on T1D, patients treated with *ex vivo* expanded polyclonal Treg cells experienced minimal to no side effects. In one trial, the infused Treg cells were labeled with deuterium, allowing their tracking in peripheral blood. The authors found that a small fraction of the infused Tregs could be detected in peripheral blood up to one year after treatment and they maintained a Treg phenotype, suggesting that there was no Treg instability (55). Encouragingly, in some of the trials there was evidence that treatment of T1D patients with Treg cells can increase β cell survival and C-peptide levels, concomitantly reducing dependence on exogenous insulin (55, 79, 80, 102). Of note, these studies were informed by pioneering studies using human Treg cells for the treatment of graft-versus-host disease (GvHD), in which safety and efficacy were demonstrated over the years (39, 51, 81, 103, 104).

Several strategies to improve on the quality and induction of Treg cells used for therapy are currently being tested. For instance, Kasahara and colleagues (105) set out to optimize the generation of stable induced Tregs (iTregs) to prevent GvHD in mice, where traditional iTregs have been shown to be ineffective. Co-culturing naïve T cells with allogeneic dendritic cells in the presence of TGF-β and retinoic acid resulted in alloantigen-specific iTregs. Interestingly, vitamin C stabilized Foxp3 expression in adoptively transplanted iTregs in a GvHD environment. Indeed, vitamin C therapy triggered active DNA demethylation, particularly at the conserved non-coding sequence 2 (CNS2) enhancer of the Foxp3 gene locus, reducing iTreg conversion to pro-inflammatory ex-Treg cells. GvHD symptoms were suppressed more effectively in vitamin C-treated iTregs than in untreated iTregs. Importantly, Vitamin C also boosted the generation of FOXP3^high^ iTreg population from human naïve T cells *in vitro*, which remained stable even when exposed to IL-6. Vitamin C treatment is thus a promising molecule in adoptive Treg cell immunotherapy (105). In another study, Kasagi and colleagues (106) focused on mice with experimental autoimmune encephalomyelitis (EAE, a model for MS) and autoimmune diabetes (nonobese diabetic – NOD mice) and identified a mechanism to create autoantigen-specific Treg cells *in vivo*. In brief, the authors used either systemic sublethal irradiation to cause immune cell death or monoclonal antibodies to deplete B and CD8^+^ T cells in animals with established autoimmune disorders, and then administered self-antigen-derived peptides. Interestingly, these peptides drove naïve CD4^+^ T cells into a Foxp3^+^ Treg cell fate instead of Teff cells. Mechanistically, apoptotic cells stimulated professional phagocytes to release TGF-β, which in turn promoted induced Treg cell generation. Strikingly, these *de novo* generated antigen-specific Treg cells reduced autoimmunity while maintaining immune responses to bacterial antigens. Independently, Sun and co-workers (107) developed a method for producing antigen-specific Treg cells by culturing murine CD4^+^ T cells with retinoic acid; adoptive transfer of these cells reversed the progression of collagen-induced arthritis (CIA) in mice by suppressing TNF-α. Further testing revealed that the Treg cells remained stable *in vivo* after infusion. Altogether, these techniques aimed at augmenting Treg cell numbers and/or function might one day be used in the treatment of autoimmunity in humans (**Table 1**).

Antigen-specific Treg cells perform better than polyclonal Treg cells in the prevention and treatment of autoimmune disease in animal models (108). This observation has catalyzed the development of various methods to generate antigen-specific Treg cells, such as overexpression of TCRs (109, 110), antigen-stimulated expansion (111, 112), and the utilization of CARs (77). Recent preclinical studies have shown the high potential of antigen-specific Treg cells in the treatment of various autoimmune diseases (83, 105–107). CAR Tregs, in particular, have received much attention for the immunotherapy of autoimmune disease (5). The first reports of engineered Tregs, in 2005, focused on EAE. Transgenic mice were generated expressing a chimeric receptor comprising an MHC complex bound to an EAE peptide, MBP_89-101_, linked to an intracellular CD3ζ chain. Adoptive transfer of engineered Tregs from these transgenic mice not only prevented MBP_89-101_-induced EAE, but also treated it one month post-induction, after epitope spreading had occurred, demonstrating the bystander suppression capacity of these cells (113). Follow-up studies by the same group elucidated that these engineered Tregs induced MBP_89-101_-specific T cells to secrete IL-10. Adoptive transfer of such non-transgenic MBP_89-101_-specific T cells prevented EAE in recipient mice, indicating that these engineered Tregs could also induce infectious tolerance (114). More than a decade after those initial mouse studies, human Tregs engineered with CARs were reported. Given the difficulties of modeling autoimmune disease in humanized mice, these studies targeted GvHD and organ transplant rejection instead. The first one, published in 2016, generated CAR Tregs redirected against HLA-A2 using a CAR comprising an extracellular domain with an anti-HLA-A2 scFv and an intracellular domain with a tandem CD28-CD3ζ signaling domain (77). In this study, HLA-A2 CAR Tregs successfully prevented xenogeneic GvHD (xGvHD) induction by co-injected HLA-A2-expressing PBMCs in NSG mice. Two subsequent studies by different groups confirmed this finding and further demonstrated the capacity of HLA-A2 CAR Tregs to suppress a mixed lymphocyte reaction (MLR) between HLA-A2-positive and HLA-A2-negative PBMCs *in vivo* (measured by mouse ear swelling) and protect HLA-A2-expressing human skin grafts from rejection by HLA-A2-negative PBMCs in NSG mice (115, 116).

The last two years have seen some engineered cell studies focused on T1D. In the NOD mouse, a different group engineered NOD CD4^+^ T cells by constitutively expressing Foxp3, making them phenotypically similar to Treg cells *in vitro*, and a CAR activated by insulin. Yet, despite being detectable up to 17 weeks post transfer *in vivo*, these “converted” insulin CAR Foxp3^+^ T cells did not prevent diabetes in NOD mice (117). Most recently, human Tregs bearing a humanized HLA-A2 CAR were shown to protect NSG mice from xGvHD when HLA-A2 was present either in the co-infused PBMCs or in the mouse host (HLA-A2 transgenic NSG mice), a closer recapitulation of the human disease (78). Moreover, these HLA-A2 CAR Tregs trafficked to HLA-A2-expressing mouse or human islets transplanted in the right kidney capsule of NSG mice rendered diabetic with streptozotocin (STZ) treatment and did not impair their function, whereas conventional T cells engineered in the same fashion rejected the transplanted islets in less than 2 weeks (78). Next steps include showing efficacious islet protection and diabetes prevention/reversal in NOD and more sophisticated human immune system (HIS) mouse models (118).

## Low-dose IL-2 administration

Low-dose IL-2 therapy can be seen as a strategy to fight various autoimmune diseases complementary to Treg cell infusion. CD25, the IL-2 receptor (IL-2R) α chain, is constitutively expressed at high levels by Treg cells. The IL-2R α chain, along with the β (CD122) and γ (CD132) chains, make up the high affinity IL-2R. In general, IL-2 is an essential factor for the growth of lymphocytes. Indeed, IL-2 has been FDA approved for use in cancer immunotherapy since the 1990s, to stimulate anti-tumor Teff cells and natural killer (NK) cells. Yet, the high doses of IL-2 required for tumor regression are commonly accompanied by severe side effects, such as vascular leak syndrome (119). In addition, IL-2 plays a particularly vital role in the division, activity, and stability of Treg cells. Importantly, unlike Teff cells, Treg cells do not secrete IL-2 and are thus fully dependent on exogenous sources of this cytokine (120–122). Encouragingly, low doses of IL-2, in the range of 0.33-4.5 x 10^6^ international units (IU), selectively enhance the development and maintenance of Treg cells without Teff cell activation. These observations led investigators to posit that low-dose IL-2 would be an effective treatment for autoimmunity by boosting Treg cells (123). Enhancing Treg: Teff balance in lupus-prone mice treated with recombinant IL-2 reduced disease progression and increased survival rate (98). In a study by Johnson and colleagues, NOD mice treated with IL-2 experienced an increase in the number and proportion of Treg cells (124). In T1D patients, administration of low dose IL-2 has no serious side effects, and only mild to moderate side effects, such as inflammatory reactions at the injection site and influenza syndrome, have been reported (125–127). Although low doses of IL-2 increase Treg cells in T1D, they also increase the number of other cells expressing IL-2 receptor, such as eosinophils and NK cells (127). Indeed, a recent study by Dong and colleagues where T1D patients were treated with autologous polyclonal Treg cells followed by one or two courses of low-dose IL-2 revealed that, while IL-2 increased the numbers of both infused and endogenous Treg cells, it also boosted subsets of NK cells and CD8^+^ T cells (84). Hence, more research is needed to corroborate the use of this strategy to overcome autoimmune diseases. With regards to GvHD, numerous investigations showed that treatment with low-dose IL-2 is a beneficial regimen for achieving tolerance and reducing the risk of GvHD (128, 129). During this therapy strategy, however, NK cells were selectively expanded (121). Low-dose recombinant IL-2 treatment to active SLE patients resulted in substantial Treg cell growth, improved Treg function in peripheral blood, and clearly decreased disease activity (130–132). In a pilot study in 37 SLE patients, low-dose IL-2 increased the number of Treg cells and subsequently reduced the SLE disease activity index scores (133). A research in primary Sjogren’s syndrome reached the same outcome, confirming the unique therapeutic impact of low-dose IL-2 on immune-related diseases (134). In summary, low-dose IL-2 treatment, while promising, requires significant investigation before it can be safely employed in clinical practice. More studies are needed to determine the safe and effective dose range, including cumulative exposure, unwanted side effects, and whether there may be long-term complications. Also required are double-blind, placebo-controlled randomized trials, as well as basic research into the underlying mechanisms of IL-2 therapy. Efforts are underway to improve low-dose IL-2 therapy specificity. In one vein, investigators are mutating IL-2, generating muteins with reduced affinity to IL-2Rβγ, thus further increasing their selectivity towards cells expressing very high levels of CD25, *i.e.* Treg cells (135). Of note, recent intriguing work in the mouse aimed at circumventing the issue of specificity of IL-2 by generating an artificial molecule, ortho-IL-2, which only binds to an artificial receptor, ortho-IL-2R, allowing for the selective maintenance and expansion of Treg cells engineered to express ortho-IL-2R (136).

## Pharmacological targeting of Treg cells in autoimmune diseases

Understanding the mechanisms behind Treg cell function and number, and the pathways regulating them has yielded promising results for autoimmune disease therapy. T cell differentiation and function is highly dependent on the function of the mammalian target of rapamycin (mTOR) signaling pathway, the major nutrient-sensing regulator of cell growth, with rapamycin being a natural inhibitor of this pathway (90, 137). Studies have demonstrated that rapamycin can improve Treg cell lineage and functional stability. This stems in part from the differential regulation of the phosphatidylinositol 3-kinase (PI3K)/AKT pathway in conventional T (Tconv) cells vs. Treg cells: Tconv cells donwregulate phosphatase and tensin homologue on chromosome 10 (PTEN), a negative regulator of the PI3K/Akt pathway, which together with mTOR controls cell growth, whereas Treg cells do not. Indeed, genetically deleting PTEN specifically in Treg cells in the mouse results in Treg cell destabilization, loss of Foxp3 expression, and Th1-driven autoimmunity (138). Moreover, mTOR defects disrupt the differentiation of naïve T cells into Th1, Th2, and Th17 cells, thus favoring naïve T cell differentiation into Treg cells (139). Hence, rapamycin-mediated inhibition of mTOR disproportionally negatively affects the growth and proliferation of Tconv cells compared with Treg cells, resulting in the inclusion of rapamycin in many Treg cell *ex vivo* expansion protocols (70). Recent studies on the use of rapamycin in model animals with a variety of autoimmune diseases, such as T1D (140), autoimmune pancreatitis (141), and SLE (142), show significant expansion of Treg cells and improvement in disease symptoms. In humans, it should be noted that rapamycin treatment in SLE patients has been shown to be safe and effective (143). With regards to IPEX (144), rapamycin restores Treg cell function, including upregulation of immune-regulating proteins such as glucocorticoid-induced TNFR-related protein (GITR). Furthermore, rapamycin treatment positively impacts the histological and clinical progression of IPEX (145).

The epigenetic regulation of T-cell-mediated immunity is also beginning to be targeted in the context of Treg cells and autoimmune disease. For instance, histone deacetylase (HDAC) inhibitors have been shown to have anti-inflammatory effects and can be potential treatments for autoimmune disease patients (146), enhancing Treg cell function of Treg cells *in vitro* and *in vivo* (147). Studies have also shown that HDAC11 inhibition augments Treg suppressive function and prolongs long-term allograft survival in mice (148). Choi and colleagues reported that administration of vorinostat, an HDAC inhibitor, considerably increased the Treg cell number and FOXP3 expression in patients with hematological malignancies who received allogeneic hematopoietic stem cell transplantation (149). Severe reduction in GvHD incidence, increased Treg cell function, and inhibition of Th17 cell differentiation are the other effects of HDAC inhibitors. Interestingly, culturing RA patient-derived PBMCs with lipopolysaccharide (LPS) and HDAC inhibitors *in vitro* resulted in a greater percentage of induced Treg cells and higher IL-10 production (150), implying that HDAC inhibitors could be effective in the treatment of RA.

## Regulatory B cells

**Figure 2:**
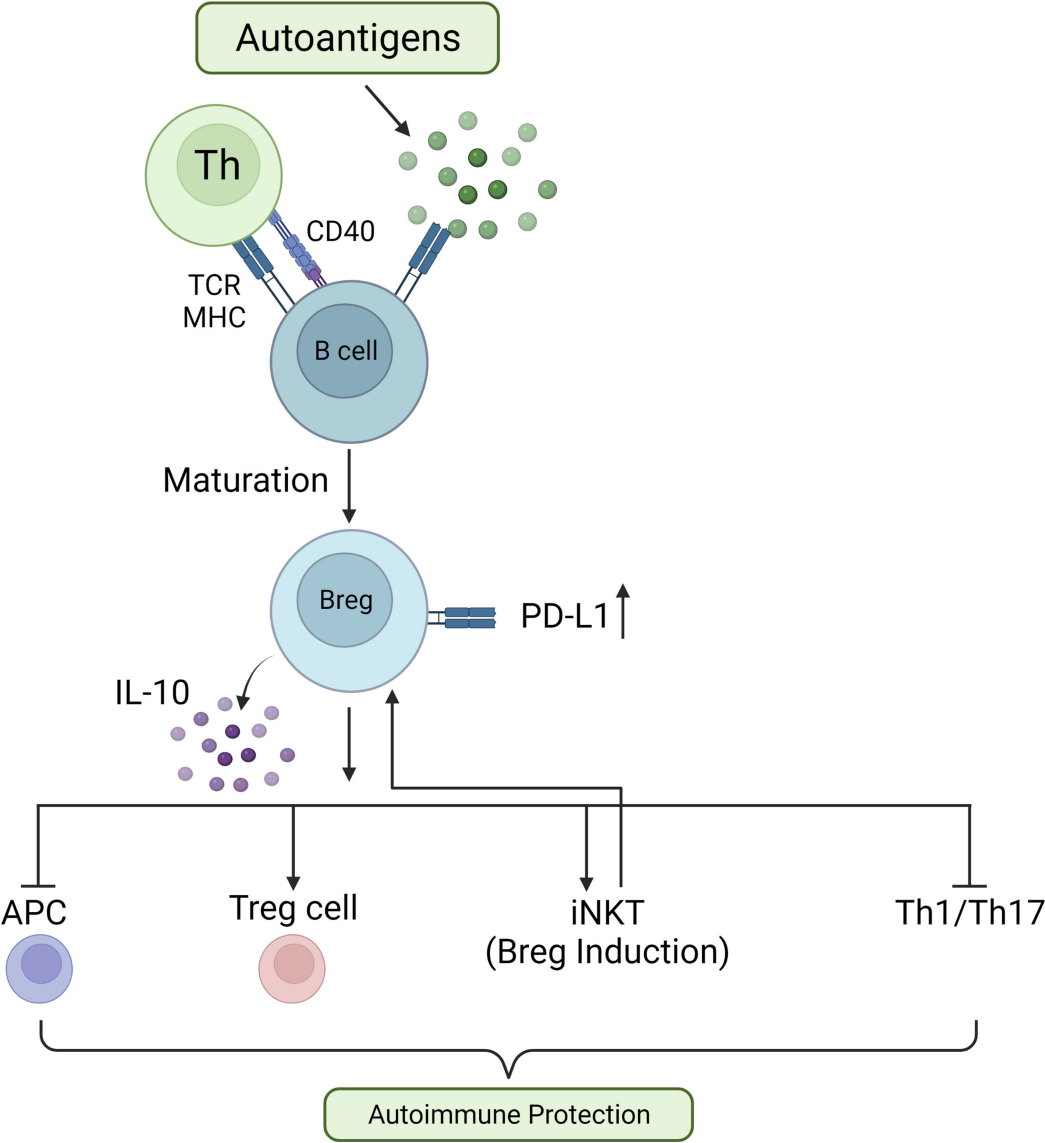
Role of Breg cells in preventing autoimmune reactions. In response to autoantigens, autoreactive B cells interact with T helper (Th) cells *via* TCR/MHC and CD40L/CD40, driving B cell differentiation into Breg cells. These IL-10-secreting Breg cells quell inflammatory responses by inhibiting APCs, increasing Treg cell activity, increasing invariant Natural Killer-like T (iNKT) cell numbers, and suppressing Th1 and Th17 cell activities.

In a complex and dynamic process, by balancing between cell division and apoptosis, B cells develop in the bone marrow and mature into functional B cells, the central players in humoral immunity (151). At the turn of the 21st century, the concept of regulatory B cells (Breg cells) was described by Bhan and Mizoguchi, referring to a subset of B cells with regulatory characteristics (152). Breg cells, similarly to Treg cells, exert their function by secreting regulatory cytokines such as TGF-β and IL-10. In addition, Breg cells can express inhibitory molecules which suppress autoreactive B cells and pathogenic T cells in a cell contact-dependent manner (153). Under the appropriate stimulation conditions and times, all types of B cells can differentiate into Breg cells. Unsurprisingly, Breg cell populations are heterogeneous (154) and the suppressive functions of Breg cells are executed through different mechanisms in different autoimmune disease models (155). In addition, it has been shown that dynamic changes in Breg cells are correlated with the development of autoimmune diseases in humans (156, 157) (**Figure 2**).

## Breg cell identification and mechanisms of action

So far, different mechanisms have been described through which Breg cells exert their impact on the immune response directly or indirectly. In mice, for example, two subsets of Breg cells have been identified. The first is a B10 cell subset (IL-10-producing B cells), characterized as CD19^hi^CD1d^hi^CD5^+^ cells, and the second is a T2-MZP cell subset (splenic transitional 2-marginal zone precursor), identified as CD19^+^CD23^+^CD21^+^CD1d^hi^ cells (158, 159). Both Breg cell subsets secrete IL-10, inhibiting T-cell proliferation and Th1 cytokine production (IFN-γ and TNF-α) (159, 160). Studies in animal models have shown that transferring a small number of *in vitro* expanded Breg cells suffices to protect the recipient animals against various autoimmune diseases for a long period of time. This suggests that Breg cells either divide *in vivo* and/or can create an efficient immunosuppressive cascade with other immunosuppressive cells (161). In addition to suppressing Th1-mediated immune responses, B cells induce the generation of regulatory Tr1 cells from Teff cells (162, 163). Gray and colleagues reported that apoptotic cells (ACs) can induce T and B cells to secrete IL-10 (163). In addition, it has been shown that IL-10 derived from B cells is essential for the induction of IL-10-secreting T cells. Breg cell function is thus critical to maintaining immune homeostasis and tolerance (164). However, Breg cells make up only 10% of the circulating B cells in a healthy human (165). Because B cell-induced suppression is primarily mediated by IL-10 production, IL-10 is the main marker used to identify Breg cells. Yet, IL-10-producing B cells can be found as part of B cell populations with distinct surface markers that perform different functions (165). These subsets include CD24^hi^CD27^+^ B cells, IL-10-producing CD24^hi^CD38^hi^ B cells, CD27^int^CD38^hi^ plasmablasts, CD19^+^TIM1^+^ B cells, and CD38^+^CD1d^+^IgM^+^CD147^+^GrB^+^ B cells. All of these cells suppress pro-inflammatory responses (156, 157, 166). Interestingly, however, only approximately 20% of B cells in each of these B cell subgroups generate IL-10. For instance, IL-10-producing B cells were found to make up 21% of CD24^hi^CD27^+^ B cells, as compared to only 1% of non-CD24^hi^CD27^+^ B cells (167). Conversely, IL-10-producing Breg cells often express different levels of CD24, CD27, and CD1d.

Breg cells inhibit Th1 cell responses and Th17 cell differentiation. In addition, they convert naïve CD4^+^ T cells into Tr1 and Treg cells (156, 168). Though IL-10 is the main cytokine involved in Breg-mediated suppression, interaction with CD80 and CD86 on the surface of Breg cells improves inhibition of Th1 responses (156, 168). According to recent research, Breg cells are involved in immune responses to infections, cancer, and autoimmune diseases. For example, Breg1 cells, characterized as CD25^hi^CD71^hi^CD73^lo^ B cells, maintain allergen tolerance by producing allergy-specific IgG4 antibodies and by suppressing allergen-specific T cell proliferation (169).

Importantly, Breg cells, similarly to other immunosuppressive cells, can arise *via* differentiation into induced Breg (iBreg) cells in response to various stimuli. T cells expressing cytotoxic T lymphocyte–associated protein 4 (CTLA-4) have been shown to promote the differentiation of a population of iBreg cells that modulate immune responses by producing TGF-β and indoleamine 2,3-dioxygenase (IDO), converting naïve T cells into TGF-β- and IL-10-producing Treg cells (170). Albeit the exact role of CTLA-4^+^ T cells in Breg differentiation is unknown, it is conceivable that this Breg cell expansion mechanism occurs *in vivo* to prevent severe inflammation. In addition, CD1d-mediated lipid antigen presentation by IL-10–producing B cells (CD24^hi^CD38^hi^ Breg cells) is critical for preserving the number and activity of invariant Natural Killer-like T (iNKT) cells, a cell subset with immunosuppressive properties (171). CD39^+^CD73^+^ Breg cells are responsible for the transition from an adenosine triphosphate (ATP) driven pro-inflammatory environment to an adenosine-induced anti-inflammatory environment. Exogenous ATP can be hydrolyzed to adenosine 5′-monophosphate (AMP) and adenosine by the concerted action of the ectonucleotidases CD39 and CD73 (172). *In vitro*, CD39^+^CD73^+^ Breg cells limit CD4^+^ and CD8^+^ T cell proliferation by producing 5′-AMP following activation with CD40L and IL-4 (172). Interestingly, IL-35 has been demonstrated to be essential to murine Breg cell-mediated suppression. Mice deficient in IL-35 specifically in B cells exhibited worse illness and enhanced resistance to *Salmonella* infection in EAE (173). In a mouse model of experimental autoimmune uveitis, IL-35-induced Breg cells halted disease progression (174). Still, a role for IL-35 in Breg cells in humans is yet to be uncovered. Altogether, Breg cells are increasingly thought to have a complex function in immunological regulation, with a rising number of inhibitory mechanisms attributed to them.

Breg cells are an IL-10 producing anti-inflammatory B cell subset. However, there are no definitive cell surface markers or lineage-defining transcription factors that define Breg cells, potentially limiting the therapeutic potential of Breg cells. Although IL-10 expression has been beneficial in identifying populations of suppressive B cells in mice and humans, many surface markers used to classify Breg cells are down- or upnregulated during inflammatory responses, resulting in inherent problems in the description of different Breg cell subsets across different experimental settings, which may account for some of the discrepancies in described Breg cell subsets. As a consequence of the variety of Breg cell subsets, identifying a Breg-cell specific transcription factor, analogous to Foxp3 in Treg cells, has been a major difficulty in Breg cell - based therapies (175). The discovery of such a chemical would assist to resolve the phenotypic of Breg cells and address the question of whether these cells constitute a different lineage. So far, two models of Breg cell formation have been proposed. The first is that, like thymus-derived Treg cells, Breg cells are a distinct lineage of B cells in which a specific factor regulates the expression of genes responsible for their suppressive character. The second is that in response to certain stimuli, B cells adopt a regulatory phenotype in order to reduce local inflammation. Despite significant effort, no study using gene arrays on Breg cells in both mice and humans has clearly discovered a lineage specific marker analogous to Foxp3 (169, 173). The inability to find a distinct transcription factor, along with the variety of the phenotypic of Breg cells, supports the hypothesis that suppressor B cells are “reactive,” rather than lineage specific. In contrast to natural Treg cells, any B cell might possibly develop into a “Breg” cell in response to the correct environmental signals (176).

## Breg cells in autoimmune disease

Abundant evidence now indicates that defects in the number and/or function of Breg cells are associated with various autoimmune diseases in mice, with severity of autoimmunity being inversely correlated with the number of Breg cells (156, 168, 171, 177, 178). Regarding autoimmune diseases, two main hypotheses are proposed for the role of Breg cells. First, inflammation is exacerbated by the lack of immunological suppression caused by Breg cells. A second possibility is that chronic inflammation is the cause of decreased Breg cell numbers and function. Currently, human studies on Breg cells are very limited, and evidence for both theories is largely derived from studies in animal models. When compared to wild-type (WT) mice, chimeric mice with an IL-10 deficiency specifically in B cells suffer from worse arthritis and EAE, accompanied by increased Th1 or Th17 cell responses, respectively (179, 180). Adoptive transfer of mouse Breg cell subgroups has also been proven to inhibit autoimmune disorders, such EAE, arthritis, and lupus (162, 180, 181). Of note, inflammatory cytokines, such as IL-6, IL-21, IL-1β, IFN-α, IFN-β and B cell-activating factor (BAFF), induce Breg cell expansion (160, 182–186). Activation of CD40 or TLR can increase this effect (182, 187, 188). Notwithstanding, chronic exposure of B cells to high doses of proinflammatory cytokines causes a decrease in functional Breg cells (182). Curiously, commensal bacteria are also involved in Breg cell expansion. In osteoarthritis mice, IL-1β and IL-6, whose secretion is influenced by the intestinal microbiota, directly increase Breg cell differentiation and IL-10 production. In agreement with a role for the microbiota, mice treated with antibiotics displayed a significant reduction in Breg cells (183).

New therapies, including those directly targeting Breg cells, are starting to address ambiguities regarding the role of Breg cells in autoimmune disease in humans. Suppressive B cells were first reported in MS patients. Helminth-infected MS patients showed a higher number of IL-10–producing CD19^+^CD1d^hi^ cells that were associated with a better clinical outcome. T cell proliferation and IFN-γ production were inhibited by B cells derived from MS patients with helminth infections (189). Although it is not easily possible to determine the exact mechanism of action in human studies, it was suggested that the disease symptoms in MS patients were reduced due to the expansion of IL-10-producing B cells – Breg cells. In addition, there is a significant decrease in IL-10-producing B cells in patients with relapsing-remitting MS (RRMS) compared with recovering patients, as well as healthy individuals (177). It is noteworthy that treatment with IFN-β for RRMS patients expands CD24^hi^CD38^hi^ Breg cells in these patients (177, 185). In EAE studies, while WT mice responded to IFN-β treatment, B cell-deficient mice did not respond to the treatment regimen, indicating that Breg cells are critical for the efficacy of IFN-β treatment also in the mouse (185). Other studies have reported the development of Breg cells in MS patients treated with alemtuzumab (an anti-CD52 therapy) and fingolimod (a sphingosine-1-phosphate modulator) (190–192). Immunomodulatory therapies in MS patients may thus operate, at least in part, by promoting a change in B cells toward an anti-inflammatory Breg cell phenotype.

Several studies on the role of Breg cells in SLE have revealed that SLE patients have defects in the number and function of circulating Breg cells, resulting from a lack of differentiation of immature CD19^+^CD24^hi^CD38^hi^ cells into Breg cells (156, 182, 193, 194). Immature B cells in SLE patients do not respond properly to known signals for Breg cell differentiation: while healthy immature B cells differentiate into Breg cells upon CD40 stimulation, CD24^hi^CD38^hi^ B cells isolated from SLE patients do not produce IL-10 following CD40 activation and are unable to suppress Th1 responses (156, 193). Moreover, SLE patients’ abundant CD19^+^FSC^hi^ polyclonally activated B cells (iBreg cells) have a considerably decreased capacity to inhibit Th cell responses when compared to the same B cell subset in healthy people (194). In addition, TLR9-activated pDCs cause a marked increase in immunosuppressive IL-10–producing CD24^+^CD38^hi^ Breg cells in healthy people, but not in SLE patients (182).

A study by Borja and colleagues demonstrated that the number of CD24^hi^CD38^hi^ Breg cells is lower in RA patients than in healthy individuals, rendering them unable to suppress Th17 cell responses and convert naïve CD4^+^ cells into Treg cells. Indeed, the number of Breg cells in RA patients is negatively correlated with the activity and severity of the disease. In the same vein, recent studies have shown that the levels of B10, IL-10^+^CD5^+^CD1d^hi^ B cells, and IL-10^+^TIM1^+^ B cells are higher in healthy people than in RA patients (168). However, in a study by Kim and colleagues (195), the number of IL-10^+^ Breg cells in RA patients was higher than in healthy individuals. The discrepancies across the studies are most likely related to the stimuli utilized to induce IL-10 production by B cells *in vitro*. The research that showed a decrease in IL-10^+^ Breg cells employed either TLR or CD40 activation of B cells, whereas the study that showed an increase used CD40 ligation in conjunction with TLR activation. More research is thus needed to consolidate the role of Breg cells in RA.

CD24^hi^CD38^hi^ B cells from individuals with pemphigus produced less IL-10 after long-term stimulation and had a worse capacity for inhibiting Th1 responses (196, 197). In contrast to untreated patients or patients who did not react to therapy, pemphigus patients who responded to treatment with the B cell-depleting agent rituximab had higher frequencies of CD24^hi^CD38^hi^ B cells and IL-10 production (198). It is possible that Breg cells can help people with rituximab-resistant pemphigus regain tolerance. IL-10-producing Breg cells are considerably decreased in frequency in individuals with CD and ulcerative colitis when compared to healthy controls (199). Lower levels of suppressive IL-10–producing Breg cells have also been linked to disease development in individuals with psoriasis (178), systemic sclerosis (200, 201), and T1D (202). While more research is needed to fully comprehend the mechanisms of Breg cell-mediated suppression in these disorders, there is enough evidence to conclude that Breg cells are numerically deficient in autoimmune diseases, a factor likely contributing to loss of immunological tolerance (203). The few clinical studies on the role of Breg cells in treating autoimmune disorders are summarized in **Table 2**.

**Table 2:** Effect of Breg cells on autoimmune symptoms.

Autoimmune disease	Breg cell types	Stage of study	Efficiency	Refs.
SLE	CD24^hi^CD38^hi^ Breg cells + rituximab	Clinical study	Improved clinical response	(182)
SLE	CD19^+^CD24^hi^CD38^hi^ Breg cells	Clinical study	Maintenance of homeostatic levels of iNKT cells.	(171)

## Breg cellular therapy for autoimmune diseases

Unlike most current drugs for immune-related diseases, which targeting the symptoms of the disease and often cause widespread toxicity in the long term, cellular immunotherapy aims to accurately target and/or modify the immune cells that lead to disease progression. As this therapeutic strategy has been effective in treating several types of cancer, scientists are commencing to utilize it to treat autoimmune diseases (204). As discussed in the previous section, Breg cells seem to play a role in modulating immune responses and fostering immune tolerance. Therefore, strategies to isolate, expand, and infuse Breg cells, or otherwise expand endogenous Breg cells, can open new windows for autoimmune disease therapy (205). B cell-depletion drugs such as rituximab, an anti-CD20 monoclonal antibody, have yielded promising results in the treatment of autoimmune diseases (206, 207). For instance, the use of rituximab for treatment of experimental autoimmune vasculitis has demonstrated that it depletes B cells by inducing B cell apoptosis, enhancing Treg cells’ immunomodulatory capacity *via* IL-10 (208). However, removing all B cells also eliminates Breg cells that suppress inflammation. Hence, there is interest in targeting specific subsets of effector B (Beff) cells or Breg cells for depletion. However, although there are surface markers that can be used alone or in combination to enrich for IL-10-producing Breg cells, their specificity is not very reliable. The lack of specific markers for Breg cells is the main challenge to realize such treatment strategy (156, 157, 166, 209).

## Regulatory dendritic cells

Dendritic cells (DCs), first described in the 1970s by Ralph Steinmann (210), are professional APCs (211). Conventional DCs stimulate naïve T cells and are often more potent APCs than macrophages or B cells. Yet, some subsets of DCs in central and peripheral lymphoid organs instead induce tolerance or antigen-specific unresponsiveness (212). These subsets of DCs are collectively known as tolerogenic or regulatory DCs (DCreg) (213). Low surface expression of major histocompatibility complex (MHC) and co-stimulatory molecules is the main feature of DCreg cells, which leads to their weak capacity to induce Teff cells. Indeed, DCreg cells’ roles are to induce autoreactive T cell anergy and Treg cell differentiation, contributing to the maintenance of immune tolerance (214). DCreg cells have been utilized to treat GvHD (215) and autoimmune diseases (216). Yet, as with Treg cell-based therapies, the lack of abundant and sustained sources of the required numbers of DCreg cells is a major obstacle to the clinical use of these cells. Therefore, recent research has focused on finding ways to produce more and purer DCreg cells (217).

## DCreg cell generation and function

As mentioned above, various DC subtypes play a vital role in maintaining immune homeostasis. The regulatory potential of DCreg cells relies on their immature status and is induced by signals from tissues (including the tumor microenvironment), apoptotic cells, and other immune cells (218). Immunosuppressive mediators, pathogenic stimuli, and genetic manipulation can also induce regulatory function in DCs. DCreg cells, although weakly, maintain the capacity to present antigen to T cells. In addition, they reduce co-stimulatory molecule expression (CD40, CD80, and CD86), as well as production of proinflammatory cytokines, such as IL-12. Simultaneously, DCreg cells increase their levels of inhibitory molecules (IDO, PD-L1, and CD95L) and anti-inflammatory cytokines (IL-10, TGF-β). Furthermore, DCreg cells are resistant to maturation signals (219). DCreg cells enhance immune tolerance through various mechanisms. These mechanisms include Treg cell generation, T cell apoptosis induction, T cell unresponsiveness induction (anergy), and inhibition of T cell responses (220).

**Figure 3:**
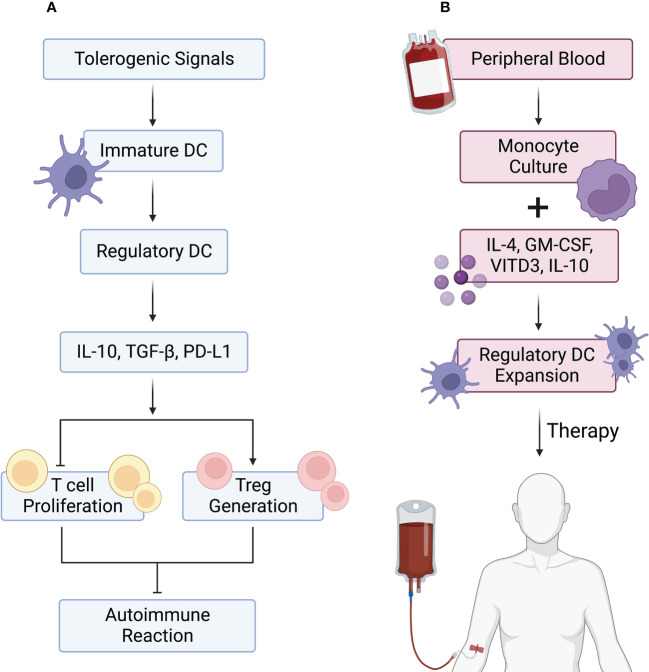
DCreg cells in autoimmune reactions and their use in autoimmune disease therapy. **(A)** Tolerogenic signals, including anti-inflammatory cytokines and apoptotic cells, lead to the differentiation of immature dendritic cells (DCs) into regulatory DCs (DCreg cells). DCreg cells are characterized by PD-L1 expression and IL-10 and TGF-β production. These properties allow DCreg cells to block effector T (Teff) cell proliferation while inducing regulatory T (Treg) cell differentiation, leading to inhibition of autoimmune reactions. **(B)** DCreg cells can be generated for therapy by isolating human peripheral blood monocytes and culturing them with IL-4, GM-CSF, vitamin D3 (vit D3), and IL-10. The resulting DCreg cells are then expanded *in vitro* and infused into patients.

Various strategies have been described to generate tolerogenic DCreg cells in mice and humans. Murine bone marrow precursors (221) and human peripheral blood monocytes (222) are the most common cell sources for DCreg cell production. Thus far, different conditions for the production of DCs have been described, with the combination of granulocyte macrophage colony-stimulating factor (GM-CSF) with IL-4 being the most frequent approach (223). *In vitro*, DC exposure to anti-inflammatory agents, either natural or pharmacological, leads to the acquisition of DCreg cell properties (**Figure 3**).

DCreg cell differentiation can be induced *in vitro* by a variety of biomolecules encountered under tolerogenic circumstances *in vivo*. In models of organ allograft rejection, allergies, and GvHD, for example, incubating DCs with IL-10 bestows them the ability to induce Treg cell generation (224, 225). Interestingly, DCs remain immature with IL-10 signaling, even in the presence of maturation signals, allowing DCs to reduce the neuropathology associated with EAE (226, 227). Treatment of human DCs with pro-inflammatory stimulants and the active form of vitamin D (1,25-dihydroxyvitamin D3) forces them to express a variety of factors key to immune tolerance induction, including TRAIL (tumor necrosis factor (TNF) receptor apoptosis-inducing ligand), IDO, IL-10, inhibitory receptors CYP24A1 and CD300LF, TGF-β, and CCL2 (228). Numerous other factors, including vasoactive intestinal peptide (VIP), thymic stromal lymphopoietin (TSLP), estrogen, GM-CSF, binding immunoglobulin protein (BiP), TNF-α, and prostaglandin (PG) E2, are involved in inducing the ability of DCreg cells to produce Treg cells (229).

The use of pharmaceutical agents has also been successful in polarizing DCs towards a DCreg cell fate both *in vitro* and in disease models (230, 231). These include histamine, anti-inflammatory agents (*e.g.* acetylsalicylic acid), adenosine receptor agonists, as well as immunosuppressive drugs, namely cyclosporine A, rapamycin, corticosteroids, BAY-117085, mycophenolatemofetil (MMF), deoxyspergualin, and tacrolimus (FK506) (232). Prednisolone or dexamethasone treatment induces the development of DCreg cells with the ability to induce Treg cells by inhibiting the expression of molecules involved in antigen presentation, inflammatory cytokines, chemokines, as well as the NF-κB pathway (233). On the other hand, rapamycin increases DCreg cells which boost Treg cell expansion *in vitro* and *in vivo* by inhibiting the mechanistic target of rapamycin (mTOR) (233, 234). DCs treated with BAY-117085, an irreversible NF-κB inhibitor, induce Treg cells and suppress established experimental autoimmune arthritis in mice (232).

Another strategy is to genetically manipulate DCs to modulate their maturity (219), with liposomes and electroporation being the most effective gene delivery methods in these cells (235). In this method, DCreg cells are produced by ectopically expressing immunomodulatory genes, including PDL-1, TGF-β, IL-4, and CTLA-4. Moreover, antisense oligodeoxy-nucleotides and small interfering RNAs (siRNAs) can be applied to silence target genes in DCs, such as CD86, IL-12, and CD40 (236, 237). In mice, these engineered DCreg cells have been demonstrated to cause T cell hyporesponsiveness, extend allograft survival, stimulate Treg cell differentiation, and decrease autoimmune diabetes or delayed-type hypersensitivity (238–240).

While several strategies for generating DCreg cells have demonstrated promising outcomes in transplantation and autoimmune disease mouse models, there are notable differences in efficacy between mice and humans. Therefore, comprehensive investigations comparing various DCreg cell-generating methodologies are required. Naranjo-Gómez and colleagues (241), for example, evaluated various factors and agents to create human DCreg cells for clinical applications and found substantial variations in DCreg cell properties, underscoring the necessity of proper agent selection. More recently, Boks and colleagues (242) examined several agents for creating therapeutic grade DCreg cells and found that IL-10-treated DCs had the highest tolerogenic potency, holding clinical potential.

## DCreg cells in autoimmune disease

As mentioned above, DCs have a dual role in immune tolerance, having both inhibitory and activating roles in autoimmune reactions (243). In the mouse, eliminating DCs leads to widespread autoimmune disease and transferring bone marrow from DC-deficient mice into WT mice leads to autoimmunity in the recipient animals, confirming the central role of DCs in maintaining immune tolerance (244). In the presence of pro-inflammatory cytokines, DCs stimulate Teff cells and prevent the production of Treg cell production. In contrast, in the presence of anti-inflammatory cytokines, DCs induce T cell anergy and induce of Treg cell generation (245). Adjusting the production of autoantibodies indirectly through cross-talk between B cells and Th cells is yet another role of DCs so that any disturbance in PRR (pattern recognition receptor) signaling or abnormal cytokine or chemokine production in DCs can shift the balance between prevention and promotion of autoimmune disease (246). Furthermore, apoptotic cells that accumulate owing to DCs’ inability to completely absorb and remove apoptotic cells might emit danger signals, such as HMGB1 (High mobility group box protein 1) and accessible autoantigens, causing inflammatory reactions in DCs and driving autoimmunity (247). Of note, defects in DC apoptosis may also lead to an increase in DC numbers and autoimmunity. Indeed, a number of molecules involved in various stages of DC maturation, activation, differentiation, and migration have emerged as prospective anti-autoimmune targets (247, 248).

Recent work has demonstrated that dysregulation of DC function can lead to the disruption of intestinal immune homeostasis and concomitantly IBD. When PRRs activate intestinal DCs in response to infectious pathogens, they activate NF-κB signaling, which results in the production of TNF-α, IL-6, -12, and -23, as well as inflammasome signaling, which results in the secretion of IL-18 and IL-1β (249). Effector cytokines activate both innate immune (NK cells, macrophages, and γδ T cells) and adaptive immune cells (Th1, Th2, and Th17) involved in intestinal inflammation and IBD pathology (250, 251). Due to complex roles of intestinal DCs in both mediating intestinal immune homeostasis and promoting the development of IBD, much effort has gone into identifying conditioning factors that drive tolerization of intestinal DCs to prevent, alleviate, or even reverse colitis. Strikingly, DCreg cells derived using vasoactive intestinal peptide (DC-VIP) dramatically reduced the severity of TNBS-induced colitis in mice on both a clinical and histopathological level. Downregulation of Th1 cell inflammatory responses and generation of IL-10-producing Treg cells were linked to the therapeutic impact of DC VIP-DCreg cell injection. In addition, DCreg cells treated with dexamethasone and vitamin D3 or pulsed with enterobacterial extract have shown therapeutic effects in preventing colitis in several animal models (252, 253).

## Human DCreg cells and their therapeutic application in autoimmune disease

Recent animal studies have enhanced our understanding of the plasticity of DCs and DCreg cells in regulating inflammation, maintaining immune homeostasis, especially in autoimmune disease. The main challenge now is to translate our knowledge of DCs in mouse models to humans so that the therapeutic potential of DCs in autoimmune diseases can be assessed. The phenotypic differences between human and mouse DC subsets, as well as limited access to human samples, makes identifying functional parallels between mouse and human DC subsets exceedingly challenging. Various subsets of DCreg cells have been identified in humans that function as immune regulators, including CD1c^+^ cDCs in the blood and liver and CD141^+^CD14^+^ DCs in human skin (254, 255). Liver CDs (CD1c^+^) show high production of IL-10 upon LPS induction and induce IL-10-producing Treg cells and IL-4-producing Th2 cells (256). Recent studies have highlighted the specificity of DCreg cells in patients with autoimmune diseases and their potential for the treatment of these diseases. DCreg cells from RRMS patients conditioned with vitamin D3 exhibited a stable semi-mature phenotype and caused stable antigen-specific T cell hyporesponsiveness (256). In a study by Harry colleagues, DCs from RA patients were conditioned with clinical-grade dexamethasone and vitamin D3, which reduced stimulation of autologous antigen-specific T cells, inhibited mature DC-induced T cell activation, and made T cells hyporesponsive to subsequent stimulation (257). In a phase 1 clinical trial in patients with T1D, autologous DC therapy was performed. These DCs were generated *ex vivo* with antisense oligonucleotides targeting CD40, CD80, and CD86. Treatment with these modified DCs increased the frequency of B220^+^CD11c^-^ B cell populations without any discernible side effects in diabetic patients (258). In another phase I trial, DCreg cells generated with NF-κB signaling inhibitors were produced in RA patients, and tolerance in these patients was increased with no serious side effects (259). These encouraging results provide prospective therapeutic methods for treating autoimmune disorders *via* injection of autologous tolerogenic DCs.

In recent years, several Phase I or I/II DCreg cell clinical trials have been concluded, with further trials currently recruiting participants or have not yet published their findings, including long-term trials in organ transplant patients (260). Because these were Phase I or I/II trials, they were designed to determine whether DCreg cell-based immunotherapy is safe and well-tolerated, rather than to test whether it is effective; the results showed that the vast great majority of patients did not experience serious side effects (258, 261–264). However, no significant illness improvement was observed in any of these protocols, even though a small subgroup of treatment group individuals in the RA (261, 265) and Crohn’s disease (262) studies reported reduced symptoms.

Despite the challenges in translating DCreg cell therapy from model animals to humans, technological innovations such as closed cell culture systems have helped to bring the use of DCreg cells into the clinic for the treatment of T1D and RA. The first clinical trial of DCreg cells was conducted in T1D patients. Autologous DCs were treated *ex vivo* with antisense oligonucleotides to reduce CD80, CD86, and CD40 expression. In this clinical trial, autologous DCreg cells were safe and did not cause side effects in T1D patients (258). In the clinical trial for RA, DCreg cells were developed by treatment with BAY11-7082 and, unlike in the T1D trial, also loaded with antigens. Improvement of clinical symptoms was observed in patients with severe RA. However, none of the participants presented serious side effects, a momentous step in the development of DCreg cell-based therapies (266, 267).

In another clinical trial (NCT01352858) for RA, monocyte-derived DCreg cells, were modified with vitamin D3 and dexamethasone and treated with TLR4 agonist and clinical-grade MPLA (Monophosphoryl lipid A) (268). The TLR4 ligand is essential for DCs to successfully process and display antigen on MHC class II molecules, and it may also give DCs the capacity to move to lymph nodes in a CCR7-dependent manner, where they can engage with T cells and induce autoantigen-specific Treg. Produced DCregs by this method show a high expression level of the MHC-II, while CD80 and 86 expression is intermediate, and the expression of CD40 is very low (268). Thus, despite the ability to present antigen, their capacity to stimulate T cells is lower than that of adult DCs. Furthermore, these DCreg cells had high TGF-β and IL-10 levels while having low IL-12, IL-23, and TNF-α levels. In a CIA mouse model, DCreg cells produced with LPS, vitamin D3, and dexamethasone greatly reduced the severity and development of arthritis (269). Human DCreg cells with the potential to suppress autoreactive T cells in the rheumatoid joint might be generated using a similar approach. DCreg cells present antigen in conjunction with low co-stimulatory molecule expression, causing memory T cells to become hyporesponsive while polarizing naïve T cells to produce anti-inflammatory cytokines (259). **Table 3** summarizes clinical research on the use of DCs in the therapy of autoimmune disorders.

**Table 3:** Clinical trials using DCs in autoimmune disease therapy.

Autoimmune disease	DCreg cell therapy	Stage of study	Patient No.	Status	Refs./Study ID
T1D	tolDCs targeting CD80, CD86, CD40	Phase I	10 participants	Completed	NCT00445913
T1D	tolDCs targeting CD80, CD86, CD40	Phase II	24 participants	Not yet recruiting	NCT02354911
T1D	tolDCs and Proinsuline-loaded VitD3-tolDCs	Phase I	10 participants	Safe and well tolerated and upregulation of B220+ CD11c− B-cell population	NTR5542
T1D	Autologous DCs (AVT001)	Phase I and II	24 participants	Active, not recruiting	NCT03895996
RA	tolDC-Dex-VitD3 loaded with autologous synovial fluid	Phase I	15 participants	Unknown	NCT01352858
RA	Dex-tolDCs	Phase I	10 participants	Completed	NCT03337165
RA	Generated DCreg cells by treating with BAY 11-7082 (Rheumavax)	Phase I	18 patients	Well tolerated, decreased in Teff cells and upregulation of Treg cells	(270)
MS	Generated Dex-tolDCs loaded with aquaporine-4-or myelin-derived peptides	Phase I	20 participants	Completed,	NCT02283671
MS	VitD3-tolDCs loaded with a pool of myelin peptides	Phase I and II	9 participants	Active, not recruiting	NCT02618902
MS	VitD3-tolDCs loaded with a pool of myelin peptides	Phase I and II	16 participants	Recruiting	NCT02903537
CD	Dex/VitA tolDCs	Phase I	–	–	2007-003469-42
CD	Dex-tolDCs	Phase I	3 participants	Terminated (low recruitment)	NCT02622763

tolDC, tolerogenic dendritic cell; Vit D3, vitamin D3; DCreg cell, regulatory dendritic cell; Dex, dexamethasone.

## Myeloid-derived suppressor cells

Myeloid-derived suppressor cells (MDSCs) were first described 30 years ago as myeloid cells with potent immunoregulatory capacity (271). Further studies have since clarified the physiological and pathological roles of MDSCs as universal immune regulators. There are two major groups of MDSCs: polymorphonuclear or granulocytic MDSCs (PMN-MDSCs), morphologically and phenotypically similar to neutrophils, and monocyte-like MDSCs, which resemble monocytes (M-MDSCs) (272). Immune suppression is one of the critical features of MDSCs, allowing these cells to be distinguishable from neutrophils and monocytes in blood (273). It has become clear that MDSCs are involved in a wide range of inflammatory disorders, including autoimmune diseases (274–276). MDSCs inhibit T cell responses, and induce Treg cells, thus playing a protective role in autoimmune diseases (277). Recently, many studies have focused on the role of MDSCs in autoimmunity. However, most of these studies were carried out using animal models and extensive investigations to explain the exact characteristics of MDSCs in autoimmune diseases in humans will need to be conducted. The role of MDSCs in suppressing the immune system shows how these cells can modulate the immune response and cause cancer cells to escape from the immune system (277). Unlike information about the role of MDSCs in tumor progression, our knowledge of their function and role in autoimmune diseases is controversial and limited. Recently, however, researchers have drawn attention to MDSCs’ role and their therapeutic potential in autoimmune diseases, such as T1D (278), SLE (275, 279), MS (280–282), RA (283) and IBD (284, 285)

## Mechanisms of action of MDSCs in autoimmunity

Hematopoietic stem cells (HSCs) in bone marrow are the source of common myeloid progenitor cells, which eventually produce immature myeloid cells (IMCs). IMCs have no suppressive activity and are immunologically inactive (286). Under normal conditions in healthy individuals, IMCs differentiate into mature and functional macrophages, DCs, and granulocytes (287). In contrast, in pathological conditions, especially in the presence of inflammation, infection, autoimmune disease, cancer or trauma, IMC differentiation into immune cells is impaired. In these circumstances, IMCs become activated, proliferate in response to external and internal factors, and differentiate into MDSCs (288). IMC expansion leads to the production of MDSCs in peripheral tissues, which inhibit immune responses by producing suppressive signals (286). Several factors, including PGE2, macrophage colony-stimulating factor (M-CSF), IL-3, cyclooxygenase-2 (COX-2), vascular endothelial growth factor (VEGF), IL-6, stem cell factor (SCF)-1, and granulocyte/macrophage colony-stimulating factor (GM-CSF) play essential roles in MDSC growth (286, 289, 290), while IFN-γ, TGF-β, IL-13, and IL-4 are linked to MDSC activation (286, 291, 292).

MDSCs use various mechanisms to suppress the immune response, based on either cell-cell contact or soluble intermediates. Induced nitric oxide synthase (iNOS) and arginase-1 (Arg-1) are enzymes whose levels are increased in MDSCs (293). L-Arginine is the common substrate of iNOS and Arg-1; iNOS catalyzes the production of nitric oxide (NO), a type of ROS, from arginine, whereas Arg-1 catalyzes the hydrolysis of L-arginine into urea and ornithine. By limiting the amounts of L-arginine, a non-essential amino acid, in the microenvironment, these enzymes restrict T cell activation and proliferation (286, 294, 295). For example, CD11b^+^Gr-1^+^ MDSC accumulation was observed in the liver of an autoimmune hepatitis (AIH) mouse model, as well as in secondary lymphatic tissues and the spleen of IBD and EAE mice (296).

Notably, in all studies, CD11b^+^Gr-1^+^ MDSCs inhibited T cell proliferation through either NO or cell-cell contact. Moreover, CD11b^+^Gr^-^1low MDSCs were seen in lupus-prone MRL Faslpr mice that developed autoimmune organ damage. *Ex vivo*, these cells suppressed CD4^+^ T cell proliferation that could be blocked by an Arg-1 inhibitor, demonstrating that Arg-1 was the primary suppressive strategy used by MDSCs in this autoimmune condition (275). Ma and colleagues recently reported that CD11b^+^Ly6C^hi^ monocytes sorted from peritoneal cells dramatically suppressed T cell proliferation ex vivo that was cell contact-dependent and involved NO and PGE2. Furthermore, these cells also limited Th1 cell differentiation while enhancing Treg cell formation in a pristane-induced lupus mouse model, indicating that these were monocytic MDSCs (276). Moreover, additional critical factors utilized by MDSCs to limit T cell proliferation and cytotoxicity include IL-10, TGF-β, COX-2, IDO, PD-L1, and PGE2 (297, 298). Of note, Treg cell-mediated suppression is also aided by MDSCs: Gr-1^+^CD115^+^ MDSCs promote Foxp3^+^ Treg cell expansion in murine tumor models (299). In addition, Ly6G^+^ phagocytic MDSCs can control cytokine secretion by B cells in the CNS, potentially playing a role in recovery rates in EAE (300). Nonetheless, further studies are required to elucidate the involvement of MDSCs in the regulation of Treg and B cells. Preclinical studies on MDSCs and their association with autoimmune disease models are summarized in **Table 4**.

**Table 4:** Preclinical studies on MDSCs in autoimmune disease.

Autoimmune disease	Mechanism of study	Animal model	Effects	Refs.
T1D	Generation of MDSCs *in vitro* and transplantation into diabetic mice	Allogeneic islet transplant into diabetic C57BL/6 mice	Treg cell proliferation induction at the allograft site and inhibition of CD8^+^ T cell responses	(301)
Proteoglycan-induced arthritis (PGIA)	Cultured MDSC-like cells in combination with GM-CSF and IL-6	PGIA in BALB/c mice	Improvement of disease symptoms after injection	(302)
IBD	CD11b+Gr-1+ MDSC therapy	VILLIN-hemagglutinin (HA) transgenic mice	Reduced T cell proliferation and increased T cell apoptosis	(285)
RA	CD11b+Gr-1+ MDSCs therapy	CIA mouse	Suppression of Th17 cell expansion and RA symptom improvement	(283)
RA	CD11b+Gr-1+ therapy (both MO-MDSCs and PMN-MDSCs)	CIA mouse	Suppression of inflammatory cytokine production and RA symptom improvement	(303)
RA	PMN-MDSCstherapy	CIA mouse	RA symptom improvement	(304)
Autoimmune hepatitis	CD11b^+^Gr1^+^ MDSC therapy	Polymicrobial sepsis in gp130 mice	Potent host-protective anti-inflammatory functions	(305)
SLE	PMN-MDSCs and M-MDSCs expanded in the spleen and kidney	(NZB x NZW)F1 mice	Improved disease symptoms	(306)
SLE	Gr-1^high^CD11b^+^ MDSC therapy	(NZB x NZW)F1 mice	Inhibition of naïve B cell differentiation	(307)
SLE	Intravenous injection of BM-derived MDSCs (CD11c-CD11b+ and Gr-1+ MDSC subsets)	roquin^san/san^ mice treated with C57BL/6 murine MDSCs	Induction of Breg cell proliferation by iNOS	(279)

SLE, systemic lupus erythematosus; T1D, type 1 diabetes; IBD, inflammatory bowel disease; RA, rheumatoid arthritis.

Generally, studying the role and function of MDSCs in mouse autoimmunity models and *in vitro* has yielded conflicting results. While MDSCs extracted from autoimmune inflammatory sites inhibit T cell responses *in vitro*, *in vivo* endogenous MDSCs fail to reduce the severity of autoimmune diseases, such as EAE (282, 308). Interestingly, however, adoptive transfer of MDSCs induces immune tolerance to self-antigens and can reduce the severity of some autoimmune diseases in mice, such as T1D (278), IBD (285), and inflammatory eye disease (309). Moreover, adoptively transferred MDSCs generated *ex vivo* inhibit GvHD and prevent graft rejection in mice (310–312). For instance, these early successes in using exogenous MDSCs to suppress autoimmunity in animal models make them a promising cell-based immunotherapy target. Sources and processes for generating exogenous MDSCs are various, with growth factors and cytokines to expand exogenous MDSCs isolated from bone marrow or peripheral blood being one such process (310, 313, 314). HSCs and even embryonic stem cells have also been used as sources of exogenous MDSCs (311).

In humans, peripheral blood-derived monocytes can be differentiated and expanded *in vitro* in the presence of PGE2 to produce large amount of functional and stable MDSCs (315). Importantly, human MDSCs generated *ex vivo* suppress CD4^+^ and CD8^+^ T cell division *via* iNOS and Arg-1 (314). These methods have the potential to be used for cell-based immunotherapy of autoimmune diseases. However, there are certain dangers connected with using MDSCs in this context. On one hand, MDSCs would not be selective for antigen-specific T cells, potentially blocking both damaging T cell responses to self-antigens and beneficial immunological responses to pathogens or malignancies. On the other hand, controlling the migration and accumulation of the injected MDSCs would also be problematic and the injection of MDSCs may result in the release of inflammatory factors. As a result, more thorough studies in animal models are required before MDSC treatment can be safely tested in humans.

## Innate lymphoid cells

Innate lymphoid cells (ILCs) are a recently unveiled group of lymphocytes which, despite their lack of TCR expression, have many parallels with CD4^+^ T helper cells. ILCs are located at barrier surfaces and respond rapidly to environmental changes by local multiplication. As with other innate immune cells, ILCs are activated before adaptive immunity develops (316). Some studies report the association and interaction of ILCs with T cells in allergy and IBD patients. Indeed, ILCs can be harmful when dysregulated, contributing to chronic inflammation and autoimmune disorders such as asthma, IBD, GvHD, psoriasis, RA, and atopic dermatitis (317).

ILCs are mainly divided into 3 subgroups according to their function, the cytokines they produce, and their dependence on different transcription factors. Group 1 ILCs, which include ILC1s and NK cells; group 2 ILCs or ILC2s, and group 3 ILCs, encompassing lymphoid tissue inducer (LTi) cells and two subsets of ILC3s, categorized as natural cytotoxicity receptor (NCR)-positive and NCR-negative ILC3s. The absence of specific markers of B, T, and other hematopoietic cell lineages is usually used to identify ILCs, in addition to differential expression of markers. For instance, CD127 is expressed on most ILC1s, but not on mature NK cells. In the same vein, ILC2s express the prostaglandin D2 receptor CRTH2 and ILC3s express the natural cytotoxicity receptor NKp44 in humans. In mice, ILC2s express the IL-33 receptor ST2, whereas ILC3s express NKp46, allowing for the distinction of these two ILC subsets (318–320).

## The role of ILCs in the pathology of autoimmune disease

To date, very few studies have implicated ILCs and their cytokines in RA (321). All three groups of ILCs have been detected in the peripheral blood of healthy individuals (322). A study by Ren and colleagues indicated that the numbers of CD3^−^CD56^+^NKp44^+^CCR6^+^ cells in peripheral blood, as well as in synovial fluid, in RA patients were increased compared to healthy people of the same age (323). In this study, it was found that CD3^−^CD56^+^NKp44^+^CCR6^+^ cells were ILC3s, and their increase is positively associated with the patients’ 28-joint disease activity score (DAS28) (323). A study by a different group confirmed an increase in ILC3 cell numbers in the synovial fluid of RA patients (324). Patients with inflammatory arthritis have also been found to have ILC1-like cells in their synovial fluid and synovial tissue; this cohort of patients included mostly RA individuals, but also patients with juvenile idiopathic arthritis and psoriatic arthritis (PsA) (325). These cells produced IFN-γ in response to a combination of IL-2, -12, and -15 stimulation (325). Interestingly, ILC1-like cells are more sensitive to IL-12 and IL-18 activation and generate more IFN than their peripheral blood counterparts (326). It is thought that these CD3^-^CD16^-^CD56^bright^ cells are either NK cells or non-cytotoxic ILC1s (327). In fact, recent research found that ILC1s predominate in the synovial fluid of RA patients (328).

## Therapeutic potential of ILCs for autoimmune disease

The discovery of cytokine pathways implicated in autoimmune disorders, as well as the identification of ILCs as prolific makers of these cytokines, suggests that these cellular actors may play a role in the pathogenesis of these diseases. The IL−23–IL−17 cytokine axis is involved in the pathogenesis of various autoimmune diseases (329). For example, the anti−IL−17 monoclonal antibody secukinumab has been used as an IL-17 inhibitor in the treatment of AS and PsA (330). Initially, anti−IL−17 monoclonal antibodies were used to target Th17 cells. However, it is now known that ILC3s are also an important source of IL-17. Therefore, ILC3s are increasingly considered as additional cellular targets for therapeutic interventions in autoimmune diseases. In addition to producing IL-17, these cells also produce other disease-promoting cytokines, including lymphotoxin, GM-CSF, IL-22, and TNF-α. Utilizing selective inhibitors of ILC3s and LTi cells in autoimmune diseases may be a useful strategy (331, 332). Tertiary lymphoid organs (TLOs), also defined as ectopic lymphoid-like structures, are temporary lymphoid cell aggregates with structural features comparable to secondary lymphoid organs. Persistent immunological activation, which can be caused by microbial infection, chronic allograft rejection, or autoimmune illness, leads to the formation of such structures (333). This strategy has been effective in animal models of T1D and arthritis (332, 334). Specific treatments based on ILCs have not yet been introduced. Anti-IL-17, anti-IL-23 or anti-LTi cells target a wide range of cells. Clarifying characteristic pathways for ILCs and research into gene expression profiles could provide researchers with more specific targets. It has been shown that CD25-specific monoclonal antibody therapy preferentially depletes ILC3s in patients with MS (335). The plasticity of ILCs (336) opens up another avenue for developing specialized treatment options to take advantage of this group of immune cells and transdifferentiate IL17-producing ILC3s into non-harmful cells.

## Conclusion

Success in treating autoimmune diseases with immune cell therapy will depend on developing treatment approaches with significant potency and effectiveness. These treatments should also have lasting effects without side effects. According to a paradigm put forward by Mous and colleagues, three aspects ought to be considered for immunotherapies to reach clinical efficacy: host immune system as the field of effect, growth factors as therapeutic enhancers, and immune cells as the effective factors. Potentially confounding biological factors, such as sex, comorbidities, age, and gut microbiota, must also be carefully evaluated given the significant variability among the human population. To modify and alter the treatment based on each patient’s unique and dynamic immunological state, frequent monitoring of effectiveness, safety, and validated biomarkers will be required. It appears that immune cell-based therapies for autoimmune diseases should be personalized according to the individual’s immune conditions and re-adjusted as the disease progresses or improves. Various types of immune regulatory cells, such as Treg cells, Breg cells, DCreg cells, MDSCs, and ILC subsets, have increasingly been the subject of extensive studies. Some of these cells, such as Treg and DCreg cells, have entered the clinical realm, while others, such as Breg cells, are in the preclinical stage and no clinical studies have yet been reported. MDSCs and ILC subsets, on the other hand, are still at the drawing board stage. However, the results obtained from applying these cell types towards autoimmune disease therapy have been promising. Continued comprehensive investigations on overcoming the challenges in using each one of these immune regulatory cell types are getting us closer to definitive treatments to several autoimmune diseases and will eliminate the suffering caused by them.

## Author contributions

The manuscript was written by all authors. RAR generated the figures. LF and ARA contributed to and revised the manuscript, which was then reviewed, commented on, and accepted by all authors. All authors contributed to the article and approved the submitted version.
